# Copper(II) Complexes of 5–Fluoro–Salicylaldehyde: Synthesis, Characterization, Antioxidant Properties, Interaction with DNA and Serum Albumins

**DOI:** 10.3390/molecules27248929

**Published:** 2022-12-15

**Authors:** Zisis Papadopoulos, Efstratia Doulopoulou, Ariadni Zianna, Antonios G. Hatzidimitriou, George Psomas

**Affiliations:** Laboratory of Inorganic Chemistry, Department of Chemistry, Aristotle University of Thessaloniki, GR-54124 Thessaloniki, Greece

**Keywords:** 5–fluoro–salicylaldehyde, copper(II) complexes, antioxidant activity, interaction with DNA, interaction with albumins

## Abstract

The synthesis, characterization and biological profile (antioxidant capacity, interaction with calf-thymus DNA and serum albumins) of five neutral copper(II) complexes of 5–fluoro–salicylaldehyde in the absence or presence of the *N*,*N’*–donor co–ligands 2,2′–bipyridylamine, 2,9–dimethyl–1,10–phenanthroline, 1,10–phenanthroline and 2,2′–bipyridine are presented herein. The compounds were characterized by physicochemical and spectroscopic techniques. The crystal structures of four complexes were determined by single-crystal X-ray crystallography. The ability of the complexes to scavenge 1,1–diphenyl–picrylhydrazyl and 2,2′–azinobis(3–ethylbenzothiazoline–6–sulfonic acid) radicals and to reduce H_2_O_2_ was investigated in order to evaluate their antioxidant activity. The interaction of the compounds with calf-thymus DNA possibly takes place *via* intercalation as suggested by UV–vis spectroscopy and DNA–viscosity titration studies and *via* competitive studies with ethidium bromide. The affinity of the complexes with bovine and human serum albumins was examined by fluorescence emission spectroscopy revealing the tight and reversible binding of the complexes with the albumins.

## 1. Introduction

Nowadays, there is no doubt that we need new drugs to counter viral pandemics, like Covid–19, super-resistant bacteria and drug-resistance for diseases like cancer [1]. After the FDA approved the medicinal administration of cisplatin in 1978, the scientific community has shown great interest in the study of bioactive metal compounds. In the literature, the main interest is focused on the study of compounds with precious and non–endogenous metals, such as platinum, gold and ruthenium. This approach has some disadvantages like the high cost at the production of the drugs and the severe side–effects of their use. A suggestion to overcome these problems is to try using less expensive endogenous metals like copper [1].

Copper is the third most abundant transition metal in the human body. It is also present in every aerobic organism. Copper is found in significant concentrations in ceruloplasmin and superoxide dismutase, which provides organisms with protection against free radicals and inflammation [2,3,4]. Inspired by nature, researchers have focused on copper complexes such as SOD-mimics, radical scavengers and anti-inflammatory agents [5,6]. On the other hand, because of the low redox potential between Cu(I) and Cu(II), copper complexes can induce cell death *via* the generation of reactive oxygen species (ROS) and act as artificial nucleases. This behavior is very useful to create compounds with antimicrobial [7], antiviral [8], anti-Alzheimer [9] and anticancer activity [10,11]. A good example of a copper compound is the anticancer drug Casiopeinas^®^, which is at the stage of clinical trials. Casiopeinas^®^ contains a mixture of copper complexes with (O–O) and (N–N) ligands and is believed to induce apoptosis *via* binding and oxidative damage to DNA [12].

**Figure 1:**
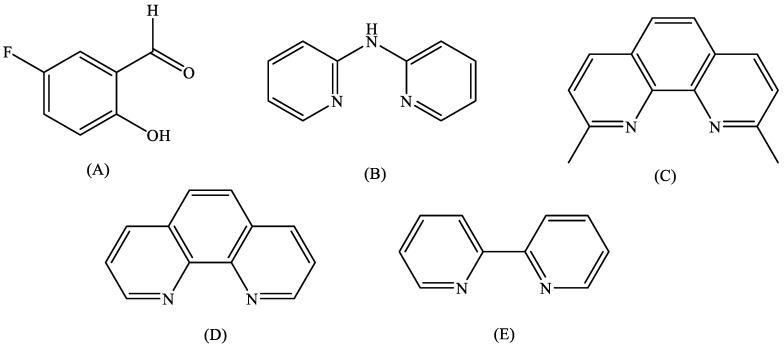
Syntax formula for (**A**) 5–fluoro–salicylaldehyde (5–F–saloH), (**B**) 2,2′–bipyridylamine (bipyam), (**C**) 2,9–dimethyl–1,10–phenanthroline (neoc), (**D**) 1,10–phenanthroline (phen) and (**E**) 2,2′–bipyridine (bipy).

Salicylaldehyde (saloH) is a natural product with oily pale–yellow color, bitter almond odor and is an ingredient of defensive secretions of some leaf beetle species [13]. Salicylaldehyde and its derivatives present interesting antimicrobial properties [14,15]. Coordination of substituted salicylaldehydes (X–saloH) on a metal may provide a wide range of biological activities to these compounds, such as DNA interaction, albumin binding, cytotoxicity, antimicrobial activity and radical scavenging ability [16,17,18,19,20,21,22,23,24,25,26]. The current research is focused on the characterization and the evaluation of the biological activity of a series of copper(II) complexes of 5–fluoro–salicylaldehyde (5–F–saloH, Figure 1A). Recent studies showed that the palladium(II) complex of 5–fluoro–salicylaldehyde presented interesting biological activity [21]. Furthermore, the choice of copper(II) was based on its versatile biological role and recent reports concerning Cu(II) with substituted salicylaldehydes, which exhibited enhanced biological profiles [15,25,26].

In the context of our ongoing research regarding metal complexes with substituted salicylaldehydes [17,18,19,20,21,22,23,24,25,26], five novel neutral copper(II) complexes of 5–F–saloH were synthesized in the absence or presence of the *N*,*N*’–donor co-ligands 2,2′–bipyridylamine (bipyam), 2,9–dimethyl–1,10–phenanthroline (neoc), 1,10–phenanthroline (phen) and 2,2′–bipyridine (bipy) (Figure 1). The complexes are formulated as [Cu(5–F–salo)_2_] (complex **1**), [Cu(5–F–salo)(bipyam)Cl] (complex **2**), [Cu(5–F–salo)(neoc)Cl].CH_3_OH (complex **3**), [Cu(5–F–salo)(phen)(NO_3_)] (complex **4**) and [Cu(5–F–salo)(bipy)(NO_3_)] (complex **5**), and were characterized by physicochemical and spectroscopic techniques, and single–crystal X-ray crystallography (the crystal structures of complexes **1**–**4** were determined). The evaluation of the biological properties of the compounds involves: (i) the potential antioxidant activity focused on the ability to scavenge 1,1–diphenyl–picrylhydrazyl (DPPH), 2,2′–azinobis(3–ethylbenzothiazoline–6–sulfonic acid) (ABTS) free radicals and to reduce H_2_O_2_, (ii) the interaction with calf-thymus (CT) DNA investigated *in vitro* by UV–vis spectroscopy, by viscosity measurements and *via* evaluating their ability to displace ethidium bromide (EB) from the DNA–EB conjugate, and (iii) the *in vitro* affinity for human serum albumin (HSA) and bovine serum albumin (BSA) was monitored by fluorescence emission spectroscopy.

## 2. Results and Discussion

### 2.1. Synthesis and Characterization

All complexes were prepared in high yields in methanolic solutions. Complex **1** was prepared from the reaction of Cu(NO_3_)_2_∙3H_2_O with deprotonated 5–fluoro–salicylaldehyde in a 1:2 ratio. The reaction of methanolic solutions of Cu(II) salts with deprotonated 5–fluoro–salicylaldehyde in the presence of the *a*–diimines bipyam, neoc, phen or bipy in a 1:1:1 ratio led to the formation of complexes **2**–**5**, respectively. Evidence of the coordination mode of the ligands in the complexes has also arisen from the interpretation of their IR and UV–vis spectra. The crystal structures of complexes **1**–**4** were further verified by single–crystal X-ray diffraction analysis.

All complexes are soluble in DMF and DMSO, but insoluble in most organic solvents and H_2_O. Molar conductivity measurements have shown that complexes **1**–**5** are non–electrolytes in DMSO solution, since the values of the Λ_M_ of the complexes in 1 mM DMSO solution were found in the range 8–12 mho∙cm^2^∙mol^−1^ [27].

The coordination of the ligands to the copper(II) ion may be confirmed by IR spectroscopy. More specifically, the broad band at 3227 cm^−1^ and the sharp one at 1381 cm^−1^, originating from the stretching and the bending vibration, respectively, of the O–H group of free 5–F–saloH, did not appear in the IR spectra of all complexes (Figure S1), confirming the successful deprotonation of the phenolate group. In addition, the shift of the band at 1271 cm^−1^ assigned to v(C_ar_–O_hydroxo_) in the spectra of complexes may indicate the binding *via* the phenolato oxygen to Cu(II). The coordination of the aldehydo oxygen can be confirmed by the shift of the band at 1663 cm^−1^ to lower wavenumbers. These features reveal the bidentate coordination of the 5–F–salo^−^ ligands to Cu(II) ion. The coexistence and the coordination of the *N*,*N*’–donors bipyam, neoc, phen and bipy may be detected by the bands at 755 cm^−1^, 732 cm^−1^, 722 cm^−1^ and 767 cm^−1^, respectively, which may be attributed to the out-of-plane vibration ρ(C_ar_–H) that is characteristic for each co-ligand [28]. For complexes **4** and **5**, the coordination of the NO_3_^−^ ligand is denoted by the presence of two characteristic vibrations at 1315 cm^−1^ and 1422–1428 cm^−1^ which are attributed to the symmetric (v_s_) and the asymmetric (v_a_) stretching vibration, respectively. The magnitude of the splitting parameter Δ (Δ = v_a_ − v_s_) is ~110 cm^−1^ and is typical of monodentate coordination (M–O–NO_2_) of nitrato ligands [29]. The suggestions from the IR spectroscopy are in good agreement with the structures determined by X-ray crystallography.

The UV–vis spectra of the complexes were recorded as nujol mull (corresponding to the solid state) and in DMSO (Figure S2) or buffer solutions used in biological experiments (150 mM NaCl and 15 mM trisodium citrate at pH values regulated in the range 6–8 by HCl solution). The spectra in nujol and DMSO did not show any appreciable differences, suggesting that the complexes keep their structure in solution [17]. In the visible region, one band appeared with λ_max_ in the range 625–750 nm which is typical for geometries expected for tetra- and penta-coordinated copper(II) complexes [30,31].

### 2.2. Structures of the Complexes

Single–crystals of complexes **1**–**4** suitable for determination of the structure by X-ray crystallography were obtained. X-ray crystallography details for complexes **1**–**4** are summarized in Table 1. For complex **5**, where single–crystals were not isolated, the structure is proposed on the basis of spectroscopic data and in comparison with the literature.

#### 2.2.1. Description of the Structure of Complex **1**

**Figure 2:**
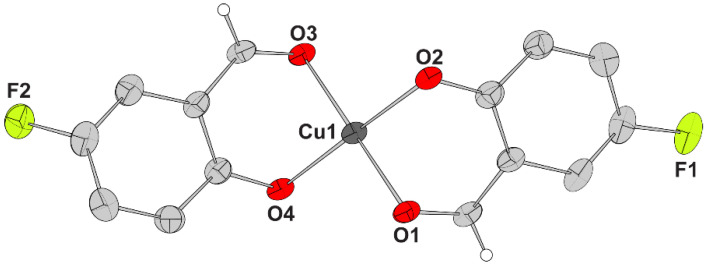
Molecular structure of complex [Cu(5–F–salo)_2_] (**1**). Aromatic hydrogen atoms are omitted for clarity.

The molecular structure of complex **1** is illustrated in Figure 2 and selected bond lengths and bond angles are given in Table 2. Complex **1** crystallized in an orthorhombic system and *Pca*2_1_ space group.

Complex **1** is a neutral mononuclear complex containing two deprotonated 5–F–salo^−^ ligands which are bound in a chelating bidentate mode to Cu(II) ion *via* the phenolato and the carbonyl oxygen atoms lying in *trans* positions. A square planar geometry around the four-coordinate copper(II) ion may be suggested based on the value of 1.71° calculated for tetrahedrality (i.e., the dihedral angle of planes formed by atoms O1, Cu1, O2 and O3, Cu1, O4, respectively; it is 0°, for strictly square planar complexes with D_4h_ symmetry, and 90° for tetrahedral complexes with D_2d_ symmetry [32]) and the values of the tetrahedral indices τ_4_ = (360° − (α + β))/(360° − 2 × 109.5°) = 0.02 [33] and τ’_4_ = ((β − α)/(360° − 109.5°)) + ((180° − β)/(180° − 109.5°)) = 0.03 [34], where β > α are the largest angles of the coordination sphere. The deviation of Cu(II) ion from the mean O_4_-plane is found to be 0.008 Å.

As expected, the Cu–O_phenolato_ lengths (1.897 (9)–1.902 (9) Å) are shorter than the Cu–O_aldehyde_ (1.928 (5)−1.932 (4) Å) lengths [17,25,26]. Complex **1** is similar to analogous square planar copper(II) complexes with X–salo^−^ ligands found in the literature [17,35,36].

#### 2.2.2. Description of the Structures of Complexes **2**–**4**

**Figure 3:**
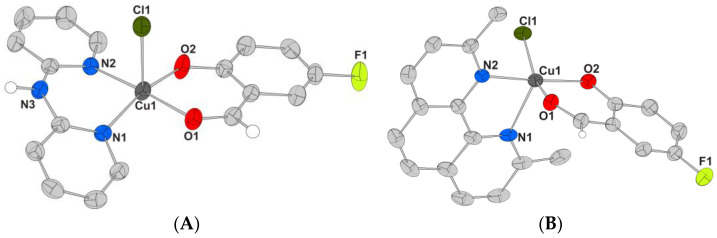

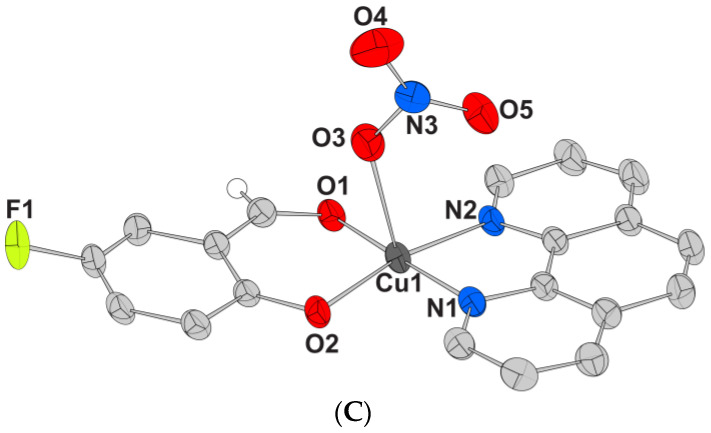
Molecular structures of (**A**) complex [Cu(5–F–salo)(bipyam)Cl] (**2**), (**B**) complex [Cu(5–F–salo)(neoc)Cl]·CH_3_OH (**3**), and (**C**) complex [Cu(5–F–salo)(phen)(NO_3_)] (**4**). For compounds **3** and **4**, only one position for each of disordered parts is shown. Aromatic and methyl hydrogen atoms and solvate molecules are omitted for clarity.

The structures of complexes **2**–**4** present similarities and differences and will be discussed together. The molecular structures of the complexes are illustrated in Figure 3 and selected bond lengths and bond angles are summarized in Table 3. Complexes **2** and **3** crystallized in a monoclinic system and *P*2_1_/*n* space group and complex **4** crystallized in a triclinic system and *P*–1 space group.

Complexes **2**–**4** are all neutral mononuclear Cu(II) complexes, having a deprotonated bidentate chelating 5–F–salo^−^ ligand coordinated to Cu(II) ion *via* its two oxygen atoms, a bidentate *α*–diimine (bipyam, neoc or phen) ligand coordinated *via* its two nitrogen atoms and a chlorido (in complexes **2** and **3**) or nitrato ligand (in complex **4**) completing the coordination sphere. A distorted square pyramidal geometry around the five–coordinated Cu(I) ions in complexes **2**–**4** may be derived *via* the values of 0.11–0.27 (Table 3) for the trigonality index τ_5_ [37], and the values of 0.79–0.92 (Table 3) for the tetragonality T^5^ [38]. The arrangement of the ligand atoms around Cu1 is not similar for all complexes: in complexes **2** and **4**, O1, O2, N1 and N2 form the basal plane and Cl1 and O3_(nitrato)_, respectively, are lying in the apical position, while in complex **3**, O1, O2, N1 and Cl1 atoms constitute the vertices of the base and N2 is on the apex.

In addition, hydrogen-bonding interactions were observed in complex **3** between the solvate methanol and O2 atom (O3—H52 = 0.82 Å, H52···O2^iv^ = 2.54 Å, O3···O2^iv^ = 3.346(10) Å, O—H52···O2^iv^ = 170° and O4—H241 = 0.82 Å, H241···O2^iv^ = 2.23 Å, O4···O2^iv^ = 3.052(10) Å, O4—H241···O2^iv^ = 180°, symmetry code: (iv) = *x* + 1/2, − *y* + 1/2, *z* − 1/2).

#### 2.2.3. Proposed Structure for Complex **5**

According to the findings of the IR and UV–vis spectroscopic data, elemental analysis and molar conductivity measurements, and after the comparison with the crystal structures of complexes **2**–**4** and with those of similar mixed-ligand copper(II) complexes found in the literature [17,39,40], we suggest that complex **5** is a mononuclear and neutral complex presenting distorted square pyramidal geometry around the penta-coordinated copper(II) ion. The 5–F–salo^−^ ligand is expected to bind in a bidentate manner to Cu(II) through the carbonyl and phenolato oxygen atoms, bipy is coordinated to Cu(II) ion through its nitrogen atoms while an oxygen atom of the monodentate nitrato ligand completes the coordination sphere.

### 2.3. Study of the Antioxidant Activity

Generally, antioxidants found mainly in food are rich in organic compounds (phenolic, hydroxyphenolic and hydroxycinnamic acids, flavones and flavonoids, etc.). The carboxylic groups in these acids or a near hydroxyl group and an oxo group for flavonoids and flavones enable them to coordinate to metal ions through their oxygen atoms leading to the formation of stable complexes. The combination of the redox properties of metal ions with such ligands is an interesting method to develop antioxidant compounds [41].

For the above reasons, the antioxidant ability of 5–F–saloH and Cu(II) complexes 1–5 has been evaluated *via* their scavenging activity towards DPPH and ABTS radicals, as well as the ability to reduce H_2_O_2_, and in comparison with that of well-known antioxidant agents such as nordihydroguaiaretic acid (NDGA), butylated hydroxytoluene (ΒHΤ), 6–hydroxy–2,5,7,8–tetramethylchromane–2–carboxylic acid (trolox) and L–ascorbic acid (these are the most commonly used standard reference antioxidant agents [42,43,44]). The results are summarized in Table 4. The DPPH-radical assay was developed in the 1950s [45] and this method has been used to assess the antioxidant capacity of several metal complexes [41]. The DPPH–scavenging ability of compounds has often been related to their ability to prevent ageing, cancer and inflammation [46]. The ability of a compound to scavenge the cationic ABTS radicals (ABTS^+●^) has been considered a measure of its total antioxidant activity [46]. Further, hydrogen peroxide has the ability to penetrate biological membranes and, although it is not very reactive itself, it can sometimes be toxic since it may give rise to hydroxyl radicals in cells. For this reason, the removal of H_2_O_2_ is very important for the protection of living systems [47]. When a compound is incubated with H_2_O_2_ using a peroxidase assay system, the loss of H_2_O_2_ can be measured [48].

Complexes **1**–**5** presented a low ability to scavenge DPPH and were found significantly less active than the reference compounds NDGA and BHT. The DPPH–scavenging ability of most complexes was found similar when incubated for 30 and for 60 min, so time did not seem to improve their action, except for complex 3 which presented enhanced DPPH–scavenging activity over time. Almost all complexes **1**–**5** can scavenge ABTS radicals more effectively than 5–F–saloH, but they are significantly less active than the reference compound trolox. Complex **1** was proved to be a much more active ABTS–scavenger (ABTS = 78.89 ± 0.18%) than the other complexes. Most complexes presented higher ability to reduce H_2_O_2_ than the reference compound L–ascorbic acid with complex **2** being the most active compound (H_2_O_2_% = 99.69 ± 0.29%). On average, complexes 1–5 presented similar or lower antioxidant activity when compared to other metal complexes with substituted salicylaldehydes as ligands [19,20,21,22].

### 2.4. Interaction of the Complexes with CT DNA

The interaction of the complexes with CT DNA was studied by UV–vis spectroscopy, viscosity measurements and *via* competitive studies with ethidium bromide. UV–vis spectroscopy may be considered a preliminary method for the study of the complexes with CT DNA, while viscosity measurements and competitive studies with EB were used to give more insight about the mode of interaction of the complexes with CT DNA, as metal complexes may interact by more than one way with DNA. In covalent binding, DNA–base nitrogen may be coordinated to metal ions after displacing at least one labile ligand of the complex. In the case of non-covalent interactions, the metal complexes interact with DNA *via* weak interactions: (i) π–π stacking interactions of the complexes between DNA base pairs (resulting in intercalation), (ii) Coulomb forces leading to electrostatic interaction outside of the helix, and (iii) van der Waals forces (hydrogen bonding, hydrophobic interactions) upon groove-binding [49].

**Figure 4:**
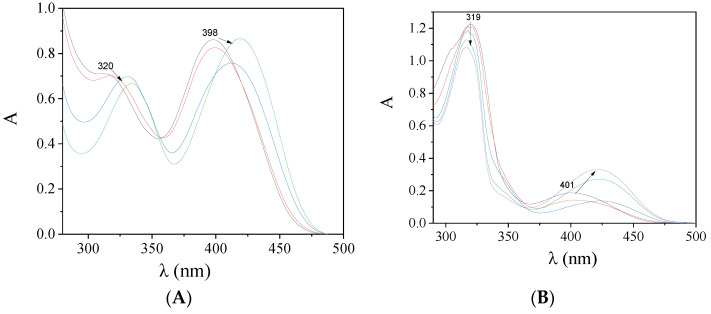

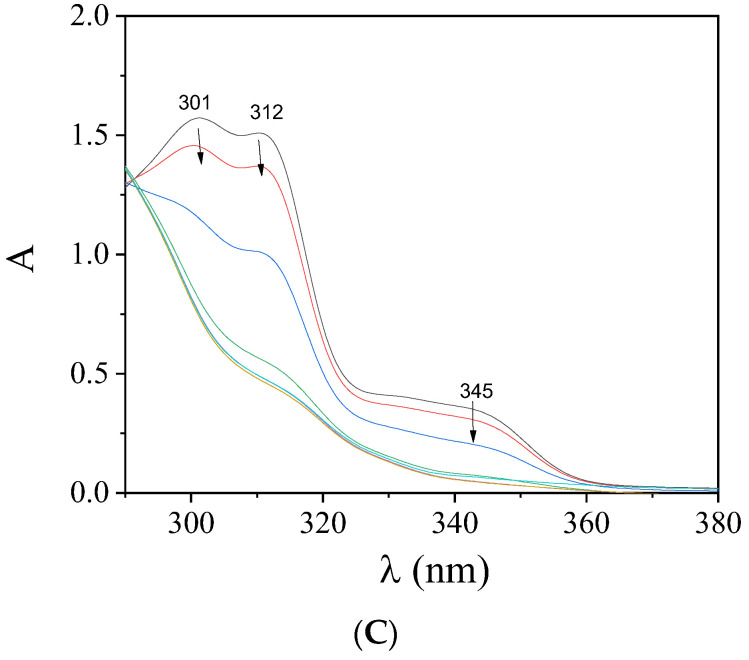
UV–vis spectra of a DMSO solution of (**A**) complex **1** (10^−4^ M), (**B**) complex **2** (5 × 10^−5^ M), and (**C**) complex **5** (10^−4^ M) in the presence of increasing amounts of CT DNA. The arrows show the changes upon increasing amounts of CT DNA.

Initially, the UV–vis spectra of complexes **1**–**5** (2.5 × 10^−5^ – 1 × 10^−4^ M) were recorded in the presence of incremental amounts of CT DNA (Figure 4), and the changes of the λ_max_ of the bands observed in the spectra of the complexes were monitored as a means to study the interaction between complexes and CT DNA [50] and to calculate the corresponding DNA-binding constants (K_b_). As observed, in the UV–vis spectra of the complexes, at least two bands were observed: band I in the range 314–339 nm and band II in the region 380–428 nm. Upon addition of the CT DNA solution, band I exhibited a significant hypochromism, and band II a rather intense hyperchromism which was mainly accompanied by a red-shift (Table 5). These features indicate the interaction of the complexes with CT DNA [51], but may not provide sufficient information to reveal the possible interaction mode. Therefore, for the elucidation of the CT DNA interaction mode, further experiments involving DNA-viscosity measurements and competitive studies with EB were performed.

The K_b_ values of compounds were calculated with the Wolfe–Shimer equation (Equation (S1)) [52] and the respective plots [DNA]/(ε_A_ − ε_f_) *versus* [DNA] revealed a tight interaction with CT DNA. The K_b_ values of complexes **1**–**5** (Table 5) were relatively high (in the order of 10^5^–10^6^ M^−1^), with complex **1** showing the highest K_b_ constant (=2.37 (±0.07) × 10^6^ M^−1^) among them and, in most cases, they are higher than the K_b_ value of the typical intercalator EB (=1.23 (±0.07) × 10^5^ M^−1^) [53]. The K_b_ values of the compounds under study are lying in the range found for analogous metal complexes of X–saloH [17,18,19,20,21,22,23,24,25,26].

**Figure 5:**
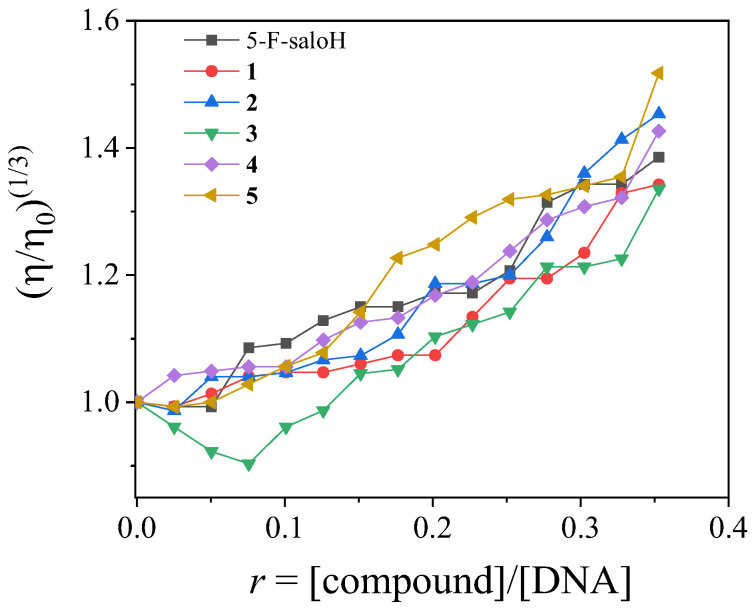
Relative viscosity (η/η_0_)^1/3^ of CT DNA (0.1 mM) in buffer solution (150 mM NaCl and 15 mM trisodium citrate at pH 7.0) in the presence of 5–F–saloH and complexes **1**–**5**, at increasing amounts (*r* = [compound]/[DNA] = 0–0.36).

The viscosity of DNA is related to the length changes occurring when interacting with a compound [54]. For this study, the viscosity of a CT DNA solution (0.1 mM) was monitored in the presence of increasing amounts (up to *r* = [compound]/[DNA] = 0.36) of the compounds at room temperature. All complexes **1**–**5** induced an increase in the relative DNA viscosity (Figure 5), which was higher in the case of complex **5**. This increase is considered evidence of an intercalative binding mode to DNA, since the DNA viscosity increases because of an increase in the separation distances between DNA bases in order to provide the necessary space for the accommodation of the intercalating compound [54].

EB is a well-known indicator of DNA intercalation, since its insertion in-between adjacent DNA base pairs may lead to the development of effective π–π stacking interactions. A solution containing the EB–DNA adduct presents an intense fluorescence emission band at 592 nm when excited at λ_ex_ = 540 nm [55]. The addition of a compound intercalating to DNA equally or more tightly than EB into this solution may induce changes to the emission band which are monitored, in order to gain insight into its competition with EB for the DNA intercalation site. The compounds under study do not present any fluorescence emission bands at RT in solution or in the presence of CT DNA or EB under the same experimental conditions; so, any changes observed in the fluorescence emission spectra of the EB–DNA solution, when the compounds are added, are useful to examine the EB–displacing ability of the complexes, as indirect evidence of their intercalating ability [55,56].

**Figure 6:**
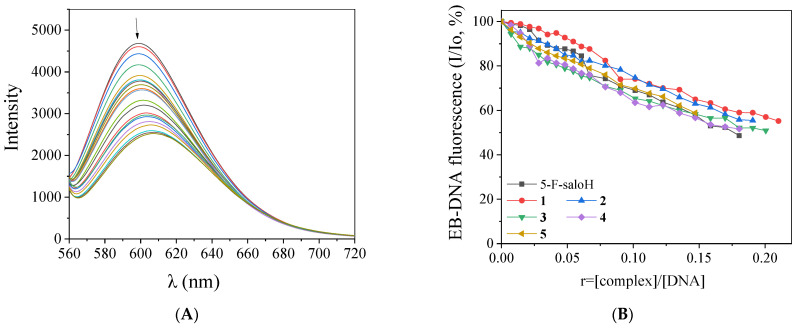
(**A**) Fluorescence emission spectra (λ_excitation_ = 540 nm) for EB–DNA conjugate ([EB] = 20 μM, [DNA] = 26 μM) in buffer solution (150 mM NaCl and 15 mM trisodium citrate at pH = 7.0) in the absence and presence of increasing amounts of complex **1** (up to *r* = 0.36). The arrow shows the changes in intensity upon increasing amounts of **1**. (**B**) Plot of EB–DNA relative fluorescence emission intensity at λ_emission_ = 592 nm (I/Io, %) *versus r* (*r* = [complex]/[DNA]) in the presence of 5–F–saloH and complexes **1**–**5** (up to 48.7% of the initial EB–DNA fluorescence emission intensity for 5–F–saloH, 55.2% for **1**, 55.5% for **2**, 50.9% for **3**, 51.6% for **4** and 58.8% for **5**).

The fluorescence emission spectra of pretreated EB–DNA ([EB] = 20 µM, [DNA] = 26 µM) were recorded in the presence of increasing amounts of the complexes (representatively shown for complex **1** in Figure 6A). The addition of the complexes resulted in a significant decrease in the intensity of fluorescence emission band of the DNA–EB compound at 592 nm (Figure 6B), with complex **5** inducing the highest quenching (Table 6). The complexes present significant ability to displace EB from the EB–DNA adduct, as it can be deducted from the observed quenching, and it can be indirectly suggested that the complexes interact with CT DNA *via* intercalation [56].

The Stern–Volmer (K_SV_) constants (Table 6) of the complexes were calculated with the Stern–Volmer equation (Equation (S2)) and Stern–Volmer plots. The K_SV_ values are relatively high (of the 10^−4^–10^−5^ M^−1^ magnitude), indicating a tight binding to CT DNA. Among the complexes, complex **1** exhibits the highest K_SV_ constant (=1.56 (±0.02) × 10^5^ M^−1^). The EB–DNA quenching constants (k_q_) of the compounds (Table 6) were calculated with Equation (S3) (considering τ_o_ = 23 ns as the EB–DNA fluorescence lifetime [57]); the k_q_ values are higher than 10^10^ M^−1^ s^−1^ [56], proposing the existence of a static quenching mechanism [21], which may confirm the interaction with the fluorophore and the displacement of EB.

### 2.5. Study of the Interaction with Serum Albumins

Serum albumins are among the important proteins of the circulatory system. Their main role is to carry drugs and other bioactive small molecules through the bloodstream [58,59]. BSA and HSA are structurally homologous albumins, having two and one tryptophan residues, respectively [60]. The tryptophan residues of both albumins are responsible for the intense fluorescence emission band with λ_em,max_ = 342 nm for BSA and 350 nm for HSA, respectively, when their solutions are excited at 295 nm [55]. The solutions of the complexes exhibited a maximum emission in the region 395–415 nm under the same experimental conditions and the SA-fluorescence emission spectra were corrected before the calculation processing. The inner-filter effect was calculated with Equation (S4) [61] and it was found too low to affect the measurements.

**Figure 7:**
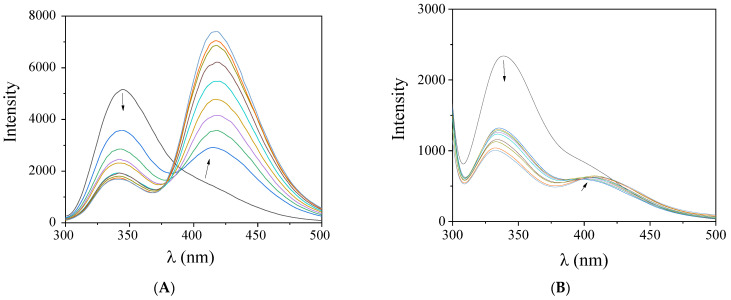
Fluorescence emission spectra (λ_excitation_ = 295 nm) of a buffer solution (150 mM NaCl and 15 mM trisodium citrate at pH 7.0) containing (**A**) BSA (3 μM) upon addition of increasing amounts of complex **5**, and (**B**) HSA (3 μM) upon addition of increasing amounts of complex **1**. The arrows show the changes in intensity upon increasing amounts of the complex.

**Figure 8:**
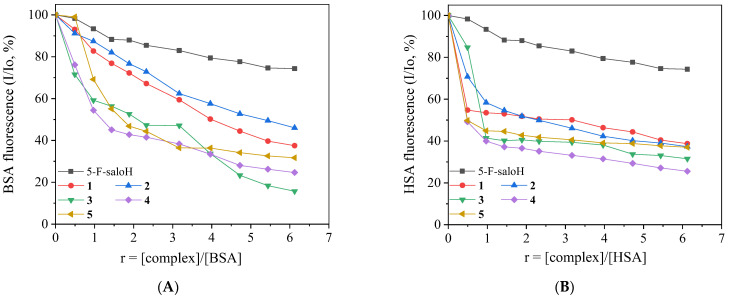
(**A**) Plot of % relative BSA fluorescence emission intensity at λ_em_ = 350 nm (I/Io, %) *versus r* (*r* = [complex]/[BSA]) for 5–F–saloH and complexes **1**–**5** (up to 74.3% of the initial BSA fluorescence for 5–F–saloH, 37.5% of **1**, 46.0% for **2**, 15.7% for **3**, 24.7% for **4**, and 31.7% for **5**) in buffer solution (150 mM NaCl and 15 mM trisodium citrate at pH 7.0). (**B**) Plot of % relative HSA fluorescence emission intensity at λ_em_ = 342 nm (I/Io, %) *versus r* (*r* = [complex]/[HSA]) for 5–F–saloH and complexes **1**–**5** (up to 74.3% of the initial HSA fluorescence for up 5–F–saloH, 38.7% for **1**, 37.3 for **2**, 31.5% for **3**, 25.5% for **4**, and 37.0% for **5**) in buffer solution (150 mM NaCl and 15 mM trisodium citrate at pH 7.0).

When the compounds were added to a solution of the albumins (3 μM), a significant quenching of the BSA (λ_em_ = 342 nm) and HSA (λ_em_ = 350 nm) fluorescence emission bands was observed (Figure 7) which was more pronounced in the case of BSA (Table 7 and Figure 8). The appearance of a second emission with band λ_max,em_ in the region 395–415 nm was attributed to the compound and, in many cases, resulted in the existence of an iso-emissive point at ~385–390 nm (Figure 7). The observed quenching may be ascribed to changes in the tryptophan environment of SA resulting from possible denaturation of their secondary structure, induced by the binding of the complexes to the albumins [62].

The SA-quenching constants (k_q_) for complexes **1**–**5** (Table 7) (calculated from the corresponding Stern–Volmer plots with the Stern–Volmer quenching equation (Equation (S2) and (S3)) are much higher than 10^10^ M^−1^ s^−1^, indicating the existence of a static quenching mechanism [56] which may indirectly verify the interaction of the compounds with the albumins. The k_q_ constants of complexes **1**–**5** are similar to those reported for similar Pd(II) and other metal complexes with substituted salicylaldehydes as ligands [12,13,20,21,22,23,24,25,26,27].

The SA-binding constants (K) of the complexes (calculated from the corresponding Scatchard plots with the Scatchard equation (Equation (S5)) (Table 7) are relatively high suggesting a tight interaction of the compounds with the albumins in order to be transported towards their potential biological targets. Furthermore, the K values are significantly lower than the value of 10^15^ M^−1^ (which is the binding constant with avidin and it is considered as the limit between reversible and irreversible interactions), suggesting a rather reversible interaction of the compounds with the albumins and revealing their ability to get released when they approach their desired destinations [63].

## 3. Materials and Methods

### 3.1. Materials—Instrumentation—Physical Measurements

All chemicals and solvents were reagent grade and were used as purchased from commercial sources: 5–F–saloH, Cu(NO_3_)_2_∙3H_2_O, CuCl_2_∙2H_2_O, CH_3_ONa, trisodium citrate, NaCl, BSA, HSA, CT DNA, EB, ABTS, K_2_S_2_O_8_, NaH_2_PO_4_, NDGA and ΒHΤ were purchased from Sigma–Aldrich Co; trolox from J&K; DPPH from TCI; L–ascorbic acid and all solvents from Chemlab.

Infrared (IR) spectra (400–4000 cm^−1^) were recorded on a Nicolet FT–IR 6700 spectrometer with samples prepared as KBr pellets (abbreviations used: s = strong, sm = strong-to-medium, and m = medium). UV–visible (UV–vis) spectra were recorded as nujol mulls and in DMSO solutions at concentrations in the range 2 × 10^−5^–5 × 10^−3^ M on a Hitachi U-2001 dual-beam spectrophotometer. C, H and N elemental analyses were performed on a PerkinElmer 240B elemental microanalyzer. Molecular conductivity measurements of 1 mM DMSO solutions of the complexes were carried out with a Crison Basic 30 conductometer. Fluorescence spectra were recorded in solution on a Hitachi F-7000 fluorescence spectrophotometer. Viscosity experiments were carried out using an ALPHA L Fungilab rotational viscometer equipped with an 18 mL LCP spindle and the measurements were performed at 100 rpm.

DNA stock solution was prepared by dilution of CT DNA with buffer (containing 150 mM NaCl and 15 mM trisodium citrate at pH 7.0) and kept at 4 °C for no longer than a week. The stock solution of CT DNA gave a ratio of UV absorbance at 260 and 280 nm (A_260_/A_280_) of 1.88, indicating that the DNA was sufficiently free of protein contamination [64]. The DNA concentration per nucleotide was determined by the UV absorbance at 260 nm after 1:20 dilution using ε = 6600 M^−1^ cm^−1^ [65].

### 3.2. Synthesis of the Complexes

#### 3.2.1. Synthesis of Complex [Cu(5–F–salo)_2_], **1**

Complex **1** was prepared according to the previously published procedure [66]. More specifically, the complex was synthesized by the addition of a methanolic solution (5 mL) of 5–F–saloH (1 mmol, 140 mg), deprotonated by CH_3_ONa (1 mmol, 54 mg) into a methanolic solution (5 mL) of Cu(NO_3_)_2_∙3H_2_O (0.5 mmol, 121 mg) at room temperature (RT). The reaction mixture was stirred for 1 h, filtered off and left to slowly evaporate. After a few days, green yellow single-crystals of complex **1** (100 mg, yield: 58%), suitable for X-ray determination were obtained. Elemental analysis: calculated for [Cu(5–F–salo)_2_], (C_14_H_8_CuF_2_O_4_) (MW = 341.76): C, 49.20; H, 2.36%; found: C, 49.15; H, 2.42%. IR spectrum (KBr), selected peaks (in cm^−1^): 1641(s), *v*(C=O); 1318 (sm), *v*(C–O → Cu); UV–vis: as nujol mull, λ/nm: 398, 695; in DMSO, λ/nm (ε/Μ^−1^ cm^−1^): 320 (7000), 398 (8500), 690 (85). Λ_M_ (in 1 mM DMSO solution) = 8 mho∙cm^2^∙mol^−1^. The complex was soluble in DMSO and DMF.

#### 3.2.2. Synthesis of Complexes **2**–**5**

The reaction of a methanolic solution of a copper(II) salt (CuCl_2_∙2H_2_O or Cu(NO_3_)_2_∙3H_2_O) (0.5 mmol) with 5–F–saloH (0.5 mmol, deprotonated by CH_3_ONa) in the presence of a methanolic solution (5 mL) of an *α*–diimine (bipyam, bipy, neoc or phen) (0.5 mmol) yielded complexes **2**–**5**. The procedure was completed by filtration and slow evaporation and afforded single-crystals for complexes **2**–**4** and microcrystalline product for complex **5**.

[Cu(5–F–salo)(bipyam)Cl], **2**: For the preparation of complex **2**, CuCl_2_∙2H_2_O (0.5 mmol, 85 mg) was the Cu(II) salt used and bipyam (0.5 mmol, 85 mg) was the corresponding *α*–diimine. Dark green single-crystals of complex **2** (105 mg, yield: 52%) suitable for X-ray determination were obtained after a week and analyzed as [Cu(5–F–salo)(bipyam)Cl], (C_17_H_13_ClCuFN_3_O_2_) (MW = 409.31): C, 49.89; H, 3.20; N, 10.27%; found: C, 49.72; H, 3.11; N, 10.15%. IR spectrum (KBr), selected peaks (in cm^−1^): 1625(s), *v*(C=O); 1327(m), *v*(C–O → Cu); 755(m), ρ(C–H)_bipyam_. UV–vis: as nujol mull, λ/nm: 405, 675 (shoulder (sh)); in DMSO, λ/nm (ε/Μ^−1^ cm^−1^): 319 (24460), 401 (3700), 680 (85). Λ_M_ (in 1 mM DMSO solution) = 10 mho∙cm^2^∙mol^−1^. The complex is soluble in DMSO and DMF and partially soluble in MeOH.

[Cu(5–F–salo)(neoc)Cl]·CH_3_OH, **3**: For the preparation of complex **3**, CuCl_2_∙2H_2_O (0.5 mmol, 85 mg) was the Cu(II) salt used and neoc (0.5 mmol, 104 mg) was the corresponding *α*–diimine. Dark green single-crystals of complex **3** (120 mg, yield: 50%) suitable for X-ray determination were obtained after ten days and analyzed as [Cu(5–F–salo)(neoc)Cl]·CH_3_OH, (C_22_H_20_ClCuFN_2_O_3_) (MW = 478.41): C, 55.23; H, 4.21; N, 5.86%; found: C, 55.11; H, 4.13; N, 5.69%. IR spectrum (KBr), selected peaks (in cm^−1^): 1610(s), *v*(C=O); 1315(m), *v*(C–O → Cu); 732(m), ρ(C–H)_neoc_. UV–vis: as nujol mull, λ/nm: 745; in DMSO, λ/nm (ε/Μ^−1^ cm^−1^): 330 (6600), 400 (4000), 750 (85). Λ_M_ (in 1 mM DMSO solution) = 12 mho∙cm^2^∙mol^−1^. The complex was soluble in DMSO and DMF and partially soluble in MeOH.

[Cu(5–F–salo)(phen)(NO_3_)], **4**: For the preparation of complex **4**, Cu(NO_3_)_2_∙3H_2_O (0.5 mmol, 121 mg) was the Cu(II) salt used and phen (0.5 mmol, 90 mg) was the corresponding *α*–diimine. Dark green single-crystals of complex **4** (115 mg, yield: 52%) suitable for X-ray determination were obtained after a fortnight and analyzed as [Cu(5–F–salo)(phen)(NO_3_)], (C_19_H_12_CuFN_3_O_5_) (MW = 444.87): C, 51.30; H, 2.72; N, 9.45%; found: C, 51.05; H, 2.59; N, 9.33%. IR spectrum (KBr), selected peaks (in cm^−1^): 1610 (s), *v*(C=O); 1428 (sm), v_a_(NO_3_); 1321(m), *v*(C–O → Cu); 1315 (sm), v_s_(NO_3_); 722 (m), ρ(C–H)_phen_. UV–vis: as nujol mull, λ/nm: 400, 655; in DMSO, λ/nm (ε/Μ^−1^ cm^−1^): 295 (sh) (5000), 330 (8700), 403 (3000), 660 (65). Λ_M_ (in 1 mM DMSO solution) = 10 mho∙cm^2^∙mol^−1^. The complex was soluble in DMSO and DMF.

[Cu(5–F–salo)(bipy)(NO_3_)], **5**: For the preparation of complex **5**, Cu(NO_3_)_2_∙3H_2_O (0.5 mmol, 121 mg) was the Cu(II) salt used and bipy (0.5 mmol, 78 mg) was the corresponding *α*–diimine. Green microcrystalline product (115 mg, yield: 55%) was precipitated after a few days and analyzed as [Cu(5–F–salo)(bipy)(NO_3_)], (C_17_H_12_CuFN_3_O_5_) (MW = 420.84): C, 48.52; H, 2.87; N, 9.98%; found: C, 48.19; H, 2.80; N, 9.73%. IR spectrum (KBr), selected peaks (in cm^−1^): 1602(s), *v*(C=O); 1422(s), v_a_(NO_3_); 1347(m), *v*(C–O → Cu); 1315 (sm), v_s_(NO_3_); 767(m), ρ(C–H)_bipy_. UV–vis: as nujol mull, λ/nm: 615; in DMSO, λ/nm (ε/Μ^−1^ cm^−1^): 312 (15000), 345 (3300), 625 (50). Λ_M_ (in 1 mM DMSO solution) = 12 mho∙cm^2^∙mol^−1^. The complex was soluble in DMSO and DMF and partially soluble in MeOH.

### 3.3. X-ray Crystal Structure Determination

Single-crystals of complexes **1**–**4** suitable for crystal structure analysis were obtained by slow evaporation of their mother liquids at RT. They were mounted at room temperature on a Bruker Kappa APEX2 diffractometer equipped with a triumph monochromator using Mo Kα (λ = 0.71073 Å, source operating at 50 kV and 30 mA) radiation. Unit cell dimensions were determined and refined by using the angular settings of at least 200 high intensity reflections (>10σ(I)) in the range 11 < 2 θ < 36°. Intensity data were recorded using φ and ω–scans. All crystals presented no decay during the data collection. The frames collected for each crystal were integrated with the Bruker SAINT Software package [67], using a narrow frame algorithm. Data were corrected for absorption using the numerical method (SADABS) based on crystal dimensions [68]. The structure was solved using the SUPERFLIP package [69], incorporated in Crystals. Data refinement (full-matrix least-squares methods on *F*^2^) and all subsequent calculations were carried out using the Crystals version 14.61 build 6236 program package [70]. All non-hydrogen non-disordered atoms were refined anisotropically. For the disordered atoms, their occupation factors under fixed (an)isotropic displacement parameters were first detected. For **3**, the methanol solvent molecule is disordered over two positions with site occupation factors of 0.5 each. The same holds for the non-coordinating nitrate oxygen atoms in complex **4**. The disordered atom positions in **3** were refined isotropically, but anisotropically in the case of **4**.

Hydrogen atoms riding on non-disordered parent atoms were located from difference Fourier maps and refined at idealized positions riding on the parent atoms with isotropic displacement parameters Uiso(H) = 1.2 Ueq(C) or 1.5 Ueq(methyl, -NH and -OH hydrogens) and at distances C--H 0.95 Å, N--H 0.83 Å and O-H 0.82 Å. All methyl, amine and OH hydrogen atoms were allowed to rotate but not to tip. Hydrogen atoms riding on disordered oxygen atoms of methanol solvent molecules were positioned geometrically to fulfill hydrogen bonding demands. The rest of the methyl hydrogen atoms were positioned geometrically to their parent atoms. Illustrations with 50% ellipsoids probability were drawn by CAMERON [71]. Crystallographic data for complexes **1**–**4** are presented in Table 1.

### 3.4. Study of the Biological Profile of the Compounds

The biological activity of the compounds (interaction with CT DNA and albumins, antioxidant activity) was evaluated *in vitro* after the compounds were dissolved in DMSO (1 mM), due to their low solubility in water. The studies were conducted in the presence of aqueous buffer solutions, where mixing of each solution never exceeded 5% DMSO (*v*/*v*) in the final solution. Control experiments were undertaken to assess the effect of DMSO on the data. Minimum or no changes were observed in the spectra of the SAs or CT DNA and appropriate corrections were performed, when needed.

All the protocols and relevant equations regarding the *in vitro* study of the biological activity (antioxidant activity, interaction with CT DNA, HSA and BSA) of the compounds can be found in the Supporting Information File (Sections S1–S3).

## 4. Conclusions

A total of five novel Cu(II) complexes of 5–fluoro–salicylaldehyde were synthesized and characterized by diverse techniques. The crystal structures of complexes **1**–**4** were determined by single crystal X-ray diffraction analysis, with complex **1** presenting square planar geometry and complexes **2**–**4** distorted square pyramidal arrangement of the ligands around Cu(II). 5–fluoro–salicylaldehyde ligands are bound in a bidentate manner to the Cu(II) ion in all complexes, *via* the carbonyl and phenolato oxygen atoms.

Complexes **1**–**5** presented a low ability to scavenge DPPH radicals, moderate ability (with the exception of complex **2**) to reduce H_2_O_2_, and had significantly high scavenging activity toward ABTS radicals, which was close to that of the reference compound trolox. The interaction of the compounds with CT DNA probably takes place *via* intercalation, as deduced by UV–vis spectroscopic, viscosity measurements and EB displacement studies, leading to a rather tight DNA binding. Furthermore, the complexes have the ability to interact strongly and reversibly with serum albumins, as well as to get released upon reaching their biotarget(s).

The herein reported results concerning the antioxidant capacity and interaction of the complexes with biomacromolecules are interesting and may lead to more specific biological studies, which could reveal pathways for further biological applications of these types of compounds.

## Figures and Tables

**Table 1:** Experimental X-ray crystallography details for complexes **1**–**4**.

	1	2	3	4
Crystal Data				
Chemical formula	C_14_H_8_CuF_2_O_4_	C_17_H_13_ClCuFN_3_O_2_	C_22_H_20_ClCuFN_2_O_3_	C_19_H_12_CuFN_3_O_5_
Moiety formula			C_21_H_16_ClCuFN_2_O_2_·CH_4_O	
*M*_r_	341.76	409.31	478.41	444.87
Crystal system	Orthorhombic	Monoclinic	Monoclinic	Triclinic
Space group	*Pca*2_1_	*P*2_1_/*n*	*P*2_1_/*n*	*P*–1
Temperature (K)	295	295	295	295
*a* (Å)	12.5045 (17)	10.3584 (4)	9.0506 (11)	7.6719 (9)
*b* (Å)	3.8457 (6)	15.4548 (8)	17.2678 (16)	9.4646 (11)
*c* (Å)	25.277 (3)	10.3842 (5)	13.4078 (15)	13.1039 (15)
α (°)	90	90	90	92.412 (3)
β (°)	90	105.4055 (16)	95.523 (3)	106.764 (4)
γ (°)	90	90	90	106.721 (4)
*V* (Å^3^)	1215.5 (3)	1602.65 (13)	2085.7 (4)	864.38 (18)
*Z*	4	4	4	2
µ (mm^−1^)	1.84	1.56	1.21	1.31
Crystal size (mm)	0.21 × 0.02 × 0.02	0.18 × 0.10 × 0.09	0.19 × 0.15 × 0.12	0.17 × 0.15 × 0.14
**Data Collection**				
Diffractometer	Bruker Kappa Apex2			
Radiation type	Mo *K*α (λ = 0.71073 Å, source operating at 50 kV and 30 mA)			
Absorption correction	Numerical, Analytical Absorption (De Meulenaer and Tompa, 1965)			
*T*_min_, *T*_max_	0.95, 0.96	0.86, 0.87	0.85, 0.86	0.81, 0.83
Measured reflections	9456	12675	18728	10790
Independent reflections	3671	3048	3968	3284
Observed reflections with [*I* > 2.0σ(*I*)]	2454	2496	3029	2964
*R*_int_	0.030	0.026	0.018	0.024
(sin θ/λ)_max_ (Å^−1^)	0.725	0.612	0.611	0.616
**Refinement**				
*R*[*F*^2^ > 2σ(*F*^2^)]	0.033	0.026	0.030	0.034
*wR*(*F*^2^)	0.066	0.052	0.056	0.054
*S*	1.00	1.00	1.00	1.00
No. of reflections	2454	2496	3029	2964
No. of parameters	191	226	269	280
No. of restraints	1	-	2	35
Δρ_max_, Δρ_min_ (e Å^−3^)	0.47, −0.51	0.21, −0.29	0.51, −0.37	0.43, −0.43
Absolute structure	Flack (1983), 1725 Friedel–pairs			
Absolute structure parameter	0.19 (2)			

**Table 2:** Selected bond lengths (Å) and angles (°) for complex **1**.

Bond	Length (Å)	Bond	Length (Å)
Cu1—O1	1.934 (2)	Cu1—O3	1.925 (2)
Cu1—O2	1.893 (4)	Cu1—O4	1.902 (4)
**Bonds**	**Angle (°)**	**Bond**	**Angle (°)**
O1—Cu1—O2	92.50 (13)	O1—Cu1—O4	86.56 (14)
O1—Cu1—O3	178.72 (14)	O2—Cu1—O4	177.6 (2)
O2—Cu1—O3	87.60 (13)	O3—Cu1—O4	93.39 (13)

**Table 3:** Selected bond lengths (Å) and angles (°) and structural parameters for complexes **2**–**4**.

	Complex 2	Complex 3	Complex 4
Bond	Length (Å)	Length (Å)	Length (Å)
Cu1—O1	1.9901 (17)	2.0471 (18)	1.9708 (16)
Cu1—O2	1.9225 (17)	1.910 (2)	1.8980 (15)
Cu1—N1	2.0151 (19)	2.247 (2)	2.007 (2)
Cu1—N2	2.0244 (19)	2.011 (2)	1.9915 (17)
Cu1—X ^1^	2.5531 (7)	2.3055 (7)	2.3672 (19)
**Bonds**	**Angles (°)**	**Angles (°)**	**Angles (°)**
Ν1—Cu1—N2	90.04 (8)	79.59 (9)	82.82 (8)
N1—Cu1—O1	88.56 (7)	95.80 (8)	175.29 (7)
N1—Cu1—O2	161.04 (8)	106.24 (9)	90.87 (7)
N1—Cu1—X ^1^	100.25 (6)	106.51 (6)	100.23 (7)
N2—Cu1—O1	176.00 (8)	85.45 (8)	93.12 (7)
N2—Cu1—O2	90.15 (7)	172.93 (9)	168.61 (8)
N2—Cu1—X ^1^	91.35 (6)	92.15 (6)	96.66 (7)
O1—Cu1—O2	89.95 (7)	89.92 (8)	92.65(12)
O1—Cu1—X ^1^	92.59 (6)	156.77 (6)	82.57 (7)
O2—Cu1—X ^1^	98.70 (6)	90.00 (6)	93.78 (7)
Trigonality index τ_5_	0.25	0.27	0.11
Tetragonality, T^5^	0.79	0.92	0.83

^1^ X = Cl1 for complexes **2** and **3**; X = O3 for complex **4**.

**Table 4:** % DPPH–scavenging ability (DPPH%), % ABTS–scavenging activity (ABTS%), and H_2_O_2_–reducing activity (H_2_O_2_ %) for 5–F–saloH and complexes 1–5.

Complex	DPPH% (30 min)	DPPH% (60 min)	ABTS%	H_2_O_2_%
5–F–saloH [21]	3.96 ± 1.16	5.56 ± 1.06	19.57 ± 0.58	71.84 ± 0.95
[Cu(5–F–salo)_2_], **1**	9.05 ± 0.54	10.79 ± 0.20	78.89 ± 0.18	71.61 ± 0.35
[Cu(5–F–salo)(bipyam)Cl], **2**	7.54 ± 0.20	7.42 ± 0.50	48.89 ± 0.38	99.69 ± 0.29
[Cu(5–F–salo)(neoc)Cl], **3**	7.42 ± 0.54	14.15 ± 0.13	46.03 ± 0.60	26.10 ± 0.66
[Cu(5–F–salo)(phen)(NO_3_)], **4**	6.15 ± 0.33	4.64 ± 0.10	48.14 ± 0.35	25.90 ± 0.76
[Cu(5–F–salo)(bipy)(NO_3_)], **5**	7.19 ± 0.26	4.06 ± 0.20	7.36 ± 0.08	69.21 ± 1.10
NDGA	87.08 ± 0.12	87.47 ± 0.12	Not tested	Not tested
BHT	61.30 ± 1.16	79.78 ± 1.12	Not tested	Not tested
Trolox	Not tested	Not tested	98.10 ± 0.48	Not tested
L–ascorbic acid	Not tested	Not tested	Not tested	60.80 ± 0.20

**Table 5:** Spectral features of the UV–vis spectra of 5–F–saloH and its complexes **1**–**5** upon addition of CT DNA. UV–band (λ_max_, in nm) (percentage of hyper-/hypo-chromism (ΔA/A_0_, %), blue-/red-shift of the λ_max_ (Δλ, in nm) and the corresponding DNA-binding constants (K_b_, M^−1^).

Compound	λ (nm) (ΔA/Aο (%) ^a^, Δλ (nm) ^b^)	K_b_ (Μ^−1^)
5–F–saloH [21]	334 (−30, +1); 421 (*>*+50,^c^ 0)	8.37 (±0.47) × 10^4^
[Cu(5–F–salo)_2_], **1**	320 (−34, +16); 398 (−20, +20)	2.37 (±0.07) × 10^6^
[Cu(5–F–salo)(bipyam)Cl], **2**	319 (−12, −3); 401 (>+50, +20)	6.35 (±0.30) × 10^5^
[Cu(5–F–salo)(neoc)Cl], **3**	334 (−41, +5); 421 (+34, +4)	2.69 (±0.45) × 10^5^
[Cu(5–F–salo)(phen)(NO_3_)], **4**	295 (−38, +4); 330 (−29, −4), 405 (+44, +19)	1.09 (±0.14) × 10^6^
[Cu(5–F–salo)(bipy)(NO_3_)], **5**	312 (−72, +3); 345 (−28, 0)	9.32 (±0.29) × 10^5^

^a^ “+” denotes hyperchromism and “−” denotes hypochromism. ^b^ “+” denotes red-shift and “−” denotes blue-shift. ^c^ “>+50” denotes intense hyperchromism.

**Table 6:** Percentage of EB–DNA fluorescence quenching (ΔI/I_0_, %), Stern–Volmer constant (K_SV_ in M^−1^) and EB–DNA quenching constant (k_q_, M^−1^ s^−1^) for 5–F–saloH and complexes **1**–**5**.

Compound	ΔΙ/Ιο (%)	K_sv_ (M^−1^)	k_q_ (M^−1^ s^−1^)
5–F–saloH [21]	51.3	3.79 (±0.11) × 10^4^	1.73 (±0.05) × 10^12^
[Cu(5–F–salo)_2_], **1**	44.8	1.56 (±0.02) × 10^5^	6.78 (±0.09) × 10^12^
[Cu(5–F–salo)(bipyam)Cl], **2**	44.5	6.23 (±0.11) × 10^4^	2.71 (±0.05) × 10^12^
[Cu(5–F–salo)(neoc)Cl], **3**	49.1	3.35 (±0.05) × 10^4^	1.46 (±0.02) × 10^12^
[Cu(5–F–salo)(phen)(NO_3_)], **4**	48.4	6.77 (±0.07) × 10^4^	2.94 (±0.03) × 10^12^
[Cu(5–F–salo)(bipy)(NO_3_)], **5**	42.2	3.14 (±0.06) × 10^4^	1.36 (±0.03) × 10^12^

**Table 7:** The quenching of the SA-fluorescence (ΔΙ/Ιο, %), the albumin-quenching (k_q_, in M^−1^ s^−1^) and albumin-binding (K, in M^−1^) constants for 5–F–saloH and complexes **1**–**5**.

Compound	ΔΙ/Ιο (%)	k_q_ (M^−1^ s^−1^)	K (M^−1^)
**BSA**			
5–F–saloH [21]	25.7	1.98 (±0.08) × 10^12^	4.31 (±0.31) × 10^4^
[Cu(5–F–salo)_2_], **1**	62.5	9.38 (±0.28) × 10^12^	4.05 (±0.02) × 10^4^
[Cu(5–F–salo)(bipyam)Cl], **2**	54.0	6.49 (±0.14) × 10^12^	3.15 (±0.18) × 10^4^
[Cu(5–F–salo)(neoc)Cl], **3**	84.3	2.75 (±0.10) × 10^13^	2.51 (±0.10) × 10^5^
[Cu(5–F–salo)(phen)(NO_3_)], **4**	75.3	1.62 (±0.06) × 10^13^	2.35 (±0.10) × 10^5^
[Cu(5–F–salo)(bipy)(NO_3_)], **5**	68.3	1.87 (±0.09) × 10^13^	3.23 (±0.12) × 10^5^
**HSA**			
5–F–saloH [21]	25.7	1.96 (±0.07) × 10^12^	4.65 (±0.35) × 10^4^
[Cu(5–F–salo)_2_], **1**	61.3	4.37 (±0.18) × 10^12^	1.58 (±0.09) × 10^5^
[Cu(5–F–salo)(bipyam)Cl], **2**	62.7	6.27 (±0.21) × 10^12^	5.09 (±0.29) × 10^5^
[Cu(5–F–salo)(neoc)Cl], **3**	68.5	1.25 (±0.05) × 10^13^	7.16 (±0.40) × 10^5^
[Cu(5–F–salo)(phen)(NO_3_)], **4**	74.6	8.63 (±0.33) × 10^12^	1.70 (±0.09) × 10^6^
[Cu(5–F–salo)(bipy)(NO_3_)], **5**	63.0	3.01 (±0.10) × 10^12^	1.31 (±0.05) × 10^6^

## Data Availability

Not applicable.

## Supplementary Materials

The following supporting information can be downloaded at: https://www.mdpi.com/article/10.3390/molecules27248929/s1. CCDC 2219048–2219051 contain the supplementary crystallographic data for this paper. These data can be obtained free of charge *via* www.ccdc.cam.ac.uk/conts/retrieving.html (or from the Cambridge Crystallographic Data Centre, 12 Union Road, Cambridge CB21EZ, UK; fax: (+44) 1223–336–033; or deposit@ccde.cam.ac.uk). Cif and checkcif files for compounds **1**–**4**. Protocols and equations regarding antioxidant activity assay (S1), binding studies with CT-DNA (S2) and albumin binding studies (S3) [72,73].

## Sample Availability

Samples of the compounds are available from the authors.

## Abbreviations

5–F–saloH = 5–fluoro–salicylaldehyde; ABTS = 2,2′–azinobis(3–ethylbenzothiazoline–6–sulfonic acid; BHT = butylated hydroxytoluene; BSA = bovine serum albumin; CT = calf–thymus; DPPH = 1,1–diphenyl–picrylhydrazyl; EB = ethidium bromide, 3,8–diamino–5–ethyl–6phenyl–phenanthridinium bromide; HSA = human serum albumin; K = SA-binding constant; K_b_ = DNA-binding constant; k_q_ = quenching constant; K_SV_ = Stern–Volmer constant; NDGA = nordihydroguaiaretic acid; R = [compound]/[DNA] ratio, or [compound]/[SA] ratio; RT = room temperature; SA = serum albumin; saloH = salicylaldehyde; trolox = 6–hydroxy–2,5,7,8–tetramethylchromane–2–carboxylic acid; X–saloH = substituted salicylaldehyde.
