# Supplementary material for: Copper(II) Complexes of 5–Fluoro–Salicylaldehyde: Synthesis, Characterization, Antioxidant Properties, Interaction with DNA and Serum Albumins
Source: Molecules. 2022 Dec 15;27(24):8929. doi: 10.3390/molecules27248929 (PMC9782626; doi:10.3390/molecules27248929)

checkCIF/PLATON report

Structure factors have been supplied for datablock(s) I

THIS REPORT IS FOR GUIDANCE ONLY. IF USED AS PART OF A REVIEW PROCEDURE FOR PUBLICATION, IT SHOULD NOT REPLACE THE EXPERTISE OF AN EXPERIENCED CRYSTALLOGRAPHIC REFEREE.

No syntax errors found. CIF dictionary Interpreting this report

Datablock: I

```
Bond precision:   C-C = 0.0067 A          Wavelength=0.71073

Cell:               a=12.5045(17)      b=3.8457(6)      c=25.277(3)
                    alpha=90           beta=90          gamma=90
Temperature:        295 K

                Calculated                     Reported
Volume          1215.5(3)                      1215.5(3)
Space group     P c a 21                       P c a 21
Hall group      P 2c -2ac                      ?
Moiety formula  C14 H8 Cu F2 O4                C14 H8 Cu1 F2 O4
Sum formula     C14 H8 Cu F2 O4                C14 H8 Cu1 F2 O4
Mr              341.75                         341.76
Dx,g cm-3       1.867                          1.867
Z               4                              4
Mu (mm-1)       1.836                          1.836
F000            684.0                          684.0
F000'           685.67
h,k,lmax        18,5,36                        18,5,36
Nref            3892[ 1987]                    3671
Tmin,Tmax       0.957,0.964                    0.950,0.960
Tmin'           0.680

Correction method= # Reported T Limits: Tmin=0.950 Tmax=0.960
AbsCorr = NUMERICAL

Data completeness= 1.85/0.94       Theta(max)= 31.023

                                                       wR2(reflections)=
R(reflections)= 0.0330( 2454)                          0.0709( 2454)

S = 1.000                    Npar= 191
```

The following ALERTS were generated. Each ALERT has the format
test-name_ALERT_alert-type_alert-level.
Click on the hyperlinks for more details of the test.

Alert level C

```
PLAT041_ALERT_1_C Calc. and Reported SumFormula    Strings  Differ     Please Check
PLAT042_ALERT_1_C Calc. and Reported MoietyFormula Strings  Differ     Please Check
PLAT341_ALERT_3_C Low Bond Precision on  C-C Bonds ...............    0.00671 Ang.
PLAT911_ALERT_3_C Missing FCF Refl Between Thmin & STh/L=    0.600          8 Report
```

Alert level G

```
PLAT066_ALERT_1_G Predicted and Reported Tmin&Tmax Range Identical          ? Check
PLAT432_ALERT_2_G Short Inter X...Y Contact  O1       ..C8       .       2.86 Ang.
                                              -1/2+x,-y,z  =      3_455 Check
PLAT432_ALERT_2_G Short Inter X...Y Contact  O3       ..C1       .       2.87 Ang.
                                              1/2+x,1-y,z  =      3_565 Check
PLAT769_ALERT_4_G CIF Embedded explicitly supplied scattering data     Please Note
PLAT808_ALERT_5_G No Parseable SHELXL Style Weighting Scheme Found     Please Check
PLAT882_ALERT_1_G No Datum for _diffrn_reflns_av_unetI/netI ......     Please Do !
PLAT912_ALERT_4_G Missing # of FCF Reflections Above STh/L=  0.600         37 Note
PLAT913_ALERT_3_G Missing # of Very Strong Reflections in FCF ....          1 Note
PLAT929_ALERT_5_G No Weight Pars,Obs and Calc R1,wR2,S not Checked          ! Info
PLAT960_ALERT_3_G Number of Intensities with I  <   - 2*sig(I) ...          1 Check
```

0 **ALERT level A** = Most likely a serious problem - resolve or explain
0 **ALERT level B** = A potentially serious problem, consider carefully
4 **ALERT level C** = Check. Ensure it is not caused by an omission or oversight
10 **ALERT level G** = General information/check it is not something unexpected

4 ALERT type 1 CIF construction/syntax error, inconsistent or missing data
2 ALERT type 2 Indicator that the structure model may be wrong or deficient
4 ALERT type 3 Indicator that the structure quality may be low
2 ALERT type 4 Improvement, methodology, query or suggestion
2 ALERT type 5 Informative message, check

It is advisable to attempt to resolve as many as possible of the alerts in all categories. Often the minor alerts point to easily fixed oversights, errors and omissions in your CIF or refinement strategy, so attention to these fine details can be worthwhile. In order to resolve some of the more serious problems it may be necessary to carry out additional measurements or structure refinements. However, the purpose of your study may justify the reported deviations and the more serious of these should normally be commented upon in the discussion or experimental section of a paper or in the "special_details" fields of the CIF. checkCIF was carefully designed to identify outliers and unusual parameters, but every test has its limitations and alerts that are not important in a particular case may appear. Conversely, the absence of alerts does not guarantee there are no aspects of the results needing attention. It is up to the individual to critically assess their own results and, if necessary, seek expert advice.

Publication of your CIF in IUCr journals

A basic structural check has been run on your CIF. These basic checks will be run on all CIFs submitted for publication in IUCr journals (*Acta Crystallographica*, *Journal of Applied Crystallography*, *Journal of Synchrotron Radiation*); however, if you intend to submit to *Acta Crystallographica Section C* or *E* or *IUCrData*, you should make sure that full publication checks are run on the final version of your CIF prior to submission.

Publication of your CIF in other journals

Please refer to the *Notes for Authors* of the relevant journal for any special instructions relating to CIF submission.

PLATON version of 28/11/2022; check.def file version of 28/11/2022

Datablock I - ellipsoid plot

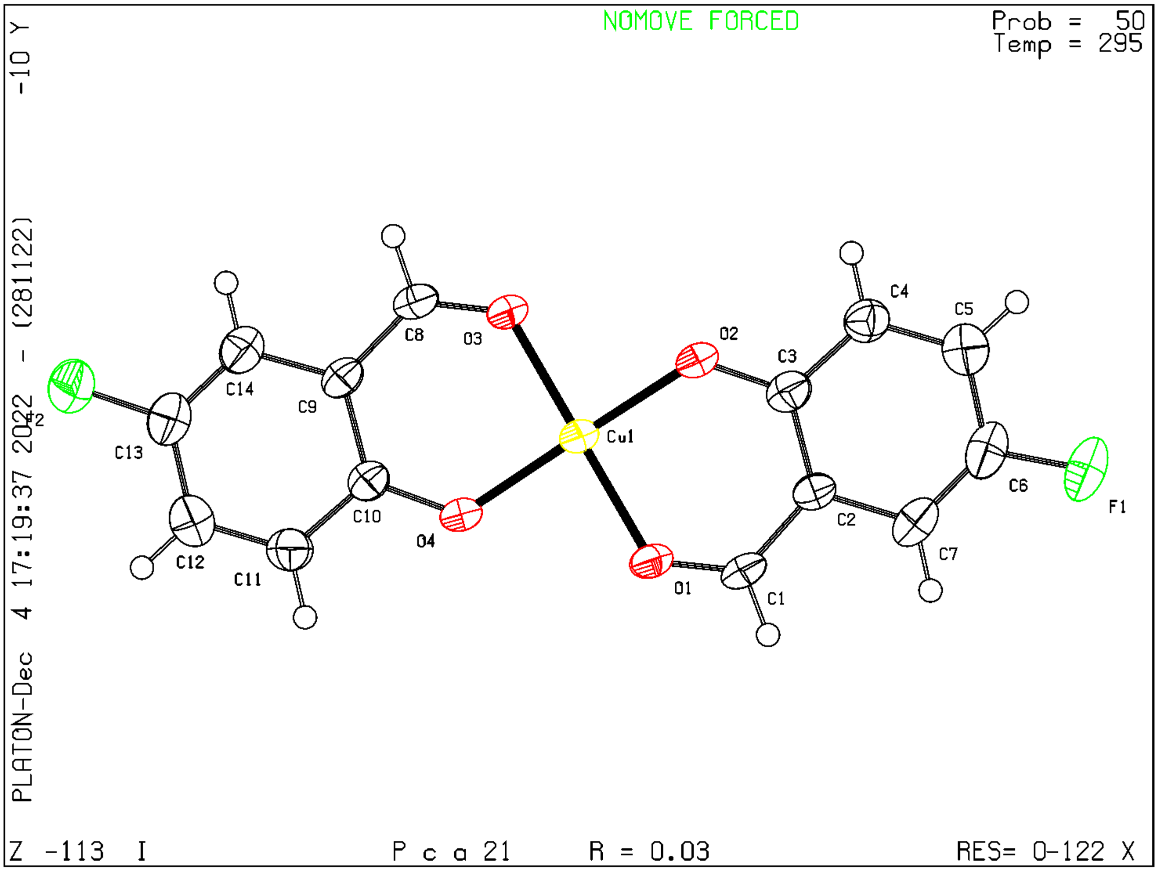

Supplement: Supplementary file 1 [file molecules-27-08929-s001.zip › checkcif of 1.pdf]
