# Supplementary material for: Copper(II) Complexes of 5–Fluoro–Salicylaldehyde: Synthesis, Characterization, Antioxidant Properties, Interaction with DNA and Serum Albumins
Source: Molecules. 2022 Dec 15;27(24):8929. doi: 10.3390/molecules27248929 (PMC9782626; doi:10.3390/molecules27248929)

checkCIF/PLATON report

Structure factors have been supplied for datablock(s) I

THIS REPORT IS FOR GUIDANCE ONLY. IF USED AS PART OF A REVIEW PROCEDURE FOR PUBLICATION, IT SHOULD NOT REPLACE THE EXPERTISE OF AN EXPERIENCED CRYSTALLOGRAPHIC REFEREE.

No syntax errors found. CIF dictionary Interpreting this report

Datablock: I

Bond precision:	C-C = 0.0042 A		Wavelength=0.71073
Cell:	a=9.0506(11)	b=17.2678(16)	c=13.4078(15)
	alpha=90	beta=95.523(3)	gamma=90
Temperature:	295 K		

	Calculated	Reported
Volume	2085.7(4)	2085.7(4)
Space group	P 21/n	P 21/n
Hall group	-P 2yn	?
Moiety formula	C21 H16 Cl Cu F N2 O2, C H4 O	C22 H20.00 Cl1 Cu1 F1 N2 O3
Sum formula	C22 H20 Cl Cu F N2 O3	C22 H20.00 Cl1 Cu1 F1 N2 O3
Mr	478.40	478.41
Dx,g cm-3	1.523	1.523
Z	4	4
Mu (mm-1)	1.210	1.210
F000	980.0	980.0
F000'	982.25	
h,k,lmax	11,21,16	11,21,16
Nref	3977	3968
Tmin,Tmax	0.804,0.865	0.850,0.860
Tmin'	0.795	

Correction method= # Reported T Limits: Tmin=0.850 Tmax=0.860
AbsCorr = NUMERICAL

Data completeness= 0.998 Theta(max)= 25.723

R(reflections)= 0.0301(3029) wR2(reflections)= 0.0588(3029)

S = 1.000 Npar= 269

The following ALERTS were generated. Each ALERT has the format
test-name_ALERT_alert-type_alert-level.
Click on the hyperlinks for more details of the test.

Alert level C

```
PLAT041_ALERT_1_C Calc. and Reported SumFormula    Strings  Differ     Please Check
PLAT042_ALERT_1_C Calc. and Reported MoietyFormula Strings  Differ     Please Check
PLAT911_ALERT_3_C Missing FCF Refl Between Thmin & STh/L=    0.600         16 Report
```

Alert level G

```
PLAT003_ALERT_2_G Number of Uiso or Uij Restrained non-H Atoms ...          4 Report
PLAT007_ALERT_5_G Number of Unrefined Donor-H Atoms .............           2 Report
PLAT300_ALERT_4_G Atom Site Occupancy of O3         Constrained at        0.5 Check
PLAT300_ALERT_4_G Atom Site Occupancy of C22        Constrained at        0.5 Check
PLAT300_ALERT_4_G Atom Site Occupancy of H31        Constrained at        0.5 Check
PLAT300_ALERT_4_G Atom Site Occupancy of H221       Constrained at        0.5 Check
PLAT300_ALERT_4_G Atom Site Occupancy of H222       Constrained at        0.5 Check
PLAT300_ALERT_4_G Atom Site Occupancy of H223       Constrained at        0.5 Check
PLAT300_ALERT_4_G Atom Site Occupancy of O4         Constrained at        0.5 Check
PLAT300_ALERT_4_G Atom Site Occupancy of C23        Constrained at        0.5 Check
PLAT300_ALERT_4_G Atom Site Occupancy of H231       Constrained at        0.5 Check
PLAT300_ALERT_4_G Atom Site Occupancy of H232       Constrained at        0.5 Check
PLAT300_ALERT_4_G Atom Site Occupancy of H233       Constrained at        0.5 Check
PLAT300_ALERT_4_G Atom Site Occupancy of H234       Constrained at        0.5 Check
PLAT302_ALERT_4_G Anion/Solvent/Minor-Residue Disorder (Resd  2  )       100% Note
PLAT302_ALERT_4_G Anion/Solvent/Minor-Residue Disorder (Resd  3  )       100% Note
PLAT769_ALERT_4_G CIF Embedded explicitly supplied scattering data     Please Note
PLAT808_ALERT_5_G No Parseable SHELXL Style Weighting Scheme Found     Please Check
PLAT860_ALERT_3_G Number of Least-Squares Restraints ............           2 Note
PLAT882_ALERT_1_G No Datum for _diffrn_reflns_av_unetI/netI ......     Please Do !
PLAT912_ALERT_4_G Missing # of FCF Reflections Above STh/L=  0.600          6 Note
PLAT929_ALERT_5_G No Weight Pars,Obs and Calc R1,wR2,S not Checked          ! Info
```

   0 **ALERT level A** = Most likely a serious problem - resolve or explain
   0 **ALERT level B** = A potentially serious problem, consider carefully
   3 **ALERT level C** = Check. Ensure it is not caused by an omission or oversight
  22 **ALERT level G** = General information/check it is not something unexpected

```
   3 ALERT type 1 CIF construction/syntax error, inconsistent or missing data
   1 ALERT type 2 Indicator that the structure model may be wrong or deficient
   2 ALERT type 3 Indicator that the structure quality may be low
  16 ALERT type 4 Improvement, methodology, query or suggestion
   3 ALERT type 5 Informative message, check
```

It is advisable to attempt to resolve as many as possible of the alerts in all categories. Often the minor alerts point to easily fixed oversights, errors and omissions in your CIF or refinement strategy, so attention to these fine details can be worthwhile. In order to resolve some of the more serious problems it may be necessary to carry out additional measurements or structure refinements. However, the purpose of your study may justify the reported deviations and the more serious of these should normally be commented upon in the discussion or experimental section of a paper or in the "special_details" fields of the CIF. checkCIF was carefully designed to identify outliers and unusual parameters, but every test has its limitations and alerts that are not important in a particular case may appear. Conversely, the absence of alerts does not guarantee there are no aspects of the results needing attention. It is up to the individual to critically assess their own results and, if necessary, seek expert advice.

Publication of your CIF in IUCr journals

A basic structural check has been run on your CIF. These basic checks will be run on all CIFs submitted for publication in IUCr journals (*Acta Crystallographica*, *Journal of Applied Crystallography*, *Journal of Synchrotron Radiation*); however, if you intend to submit to *Acta Crystallographica Section C* or *E* or *IUCrData*, you should make sure that full publication checks are run on the final version of your CIF prior to submission.

Publication of your CIF in other journals

Please refer to the *Notes for Authors* of the relevant journal for any special instructions relating to CIF submission.

PLATON version of 18/11/2022; check.def file version of 18/11/2022

Datablock I - ellipsoid plot

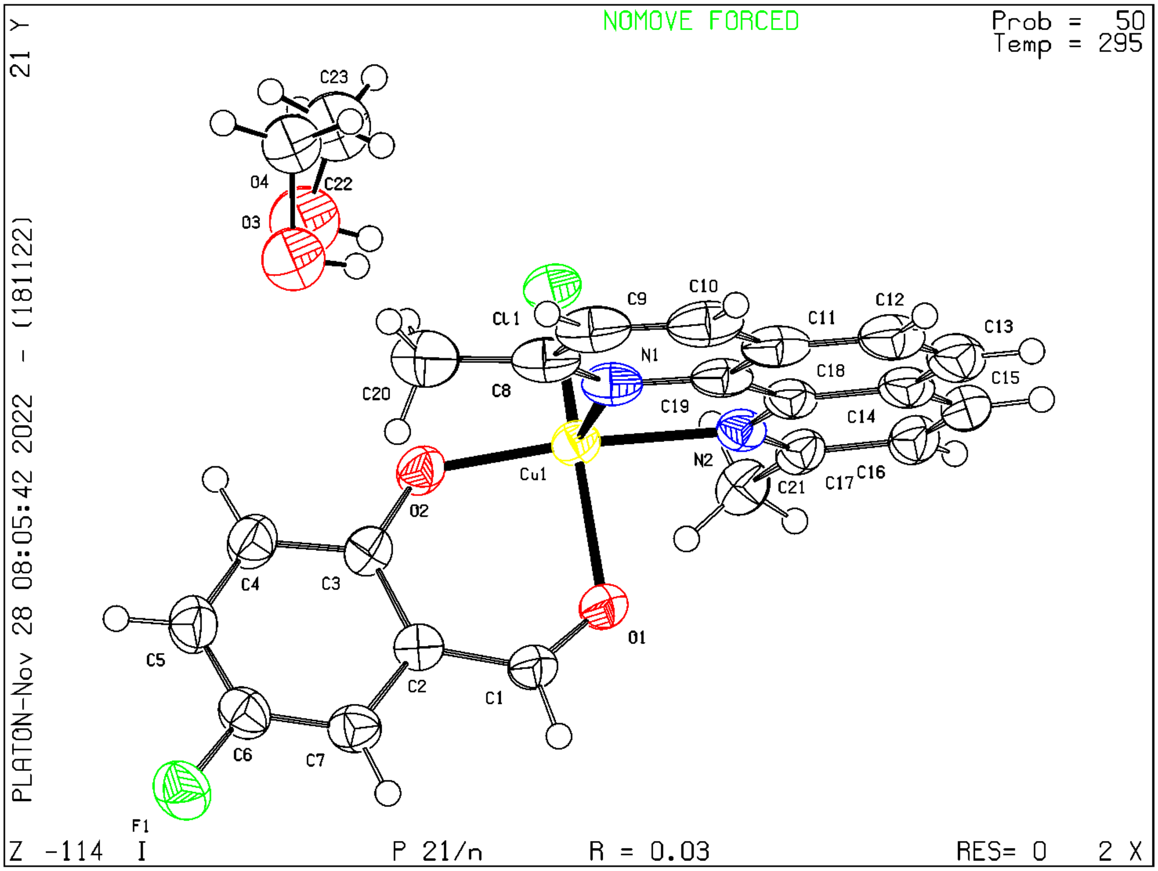

Supplement: Supplementary file 1 [file molecules-27-08929-s001.zip › checkcif of 3.pdf]
