# Supplementary material for: Copper(II) Complexes of 5–Fluoro–Salicylaldehyde: Synthesis, Characterization, Antioxidant Properties, Interaction with DNA and Serum Albumins
Source: Molecules. 2022 Dec 15;27(24):8929. doi: 10.3390/molecules27248929 (PMC9782626; doi:10.3390/molecules27248929)

checkCIF/PLATON report

Structure factors have been supplied for datablock(s) I

THIS REPORT IS FOR GUIDANCE ONLY. IF USED AS PART OF A REVIEW PROCEDURE FOR PUBLICATION, IT SHOULD NOT REPLACE THE EXPERTISE OF AN EXPERIENCED CRYSTALLOGRAPHIC REFEREE.

No syntax errors found.   CIF dictionary   Interpreting this report

Datablock: I

```
Bond precision:   C-C = 0.0036 A          Wavelength=0.71073

Cell:           a=7.6719(9)       b=9.4646(11)      c=13.1039(15)
                alpha=92.412(3)   beta=106.764(4)   gamma=106.721(3)
Temperature:    295 K
```

	Calculated	Reported
Volume	864.38(18)	864.38(18)
Space group	P -1	P -1
Hall group	-P 1	?
Moiety formula	C38 H24 Cu2 F2 N6 O10	C19 H12 Cu1 F1 N3 O5
Sum formula	C38 H24 Cu2 F2 N6 O10	C19 H12 Cu1 F1 N3 O5
Mr	889.73	444.87
Dx,g cm-3	1.709	1.709
Z	1	2
Mu (mm-1)	1.314	1.314
F000	450.0	450.0
F000'	450.85	
h,k,lmax	9,11,16	9,11,16
Nref	3387	3284
Tmin,Tmax	0.800,0.832	0.810,0.830
Tmin'	0.800	

```
Correction method= # Reported T Limits: Tmin=0.810 Tmax=0.830
AbsCorr = NUMERICAL

Data completeness= 0.970          Theta(max)= 25.971

R(reflections)= 0.0341( 2964)                  wR2(reflections)= 0.0561( 2964)

S = 1.000                  Npar= 280
```

The following ALERTS were generated. Each ALERT has the format
test-name_ALERT_alert-type_alert-level.
Click on the hyperlinks for more details of the test.

Alert level C

```
PLAT041_ALERT_1_C Calc. and Reported SumFormula    Strings  Differ     Please Check
PLAT042_ALERT_1_C Calc. and Reported MoietyFormula Strings  Differ     Please Check
PLAT241_ALERT_2_C High   'MainMol' Ueq as Compared to Neighbors of         O3 Check
PLAT242_ALERT_2_C Low    'MainMol' Ueq as Compared to Neighbors of         N3 Check
PLAT911_ALERT_3_C Missing FCF Refl Between Thmin & STh/L=    0.600         57 Report
PLAT913_ALERT_3_C Missing # of Very Strong Reflections in FCF ....         23 Note
```

Alert level G

```
PLAT002_ALERT_2_G Number of Distance or Angle Restraints on AtSite          4 Note
PLAT003_ALERT_2_G Number of Uiso or Uij Restrained non-H Atoms ...          5 Report
PLAT045_ALERT_1_G Calculated and Reported Z Differ by a Factor ...      0.500 Check
PLAT230_ALERT_2_G Hirshfeld Test Diff for    O4       --N3       .        6.2 s.u.
PLAT300_ALERT_4_G Atom Site Occupancy of O4         Constrained at        0.5 Check
PLAT300_ALERT_4_G Atom Site Occupancy of O5         Constrained at        0.5 Check
PLAT300_ALERT_4_G Atom Site Occupancy of O6         Constrained at        0.5 Check
PLAT300_ALERT_4_G Atom Site Occupancy of O7         Constrained at        0.5 Check
PLAT301_ALERT_3_G Main Residue  Disorder ..............(Resd  1  )         7% Note
PLAT432_ALERT_2_G Short Inter X...Y Contact  O4       ..C10      .       2.97 Ang.
                                                   x,-1+y,z  =      1_545 Check
PLAT432_ALERT_2_G Short Inter X...Y Contact  O6       ..C17      .       2.97 Ang.
                                                    1+x,y,z  =      1_655 Check
PLAT769_ALERT_4_G CIF Embedded explicitly supplied scattering data     Please Note
PLAT808_ALERT_5_G No Parseable SHELXL Style Weighting Scheme Found     Please Check
PLAT860_ALERT_3_G Number of Least-Squares Restraints ............          35 Note
PLAT882_ALERT_1_G No Datum for _diffrn_reflns_av_unetI/netI ......     Please Do !
PLAT910_ALERT_3_G Missing # of FCF Reflection(s) Below Theta(Min).          1 Note
PLAT912_ALERT_4_G Missing # of FCF Reflections Above STh/L=  0.600         51 Note
PLAT929_ALERT_5_G No Weight Pars,Obs and Calc R1,wR2,S not Checked          ! Info
PLAT940_ALERT_3_G Fsqd Refinement With I > n * Sigma(I) Only .....     Please Check
PLAT960_ALERT_3_G Number of Intensities with I  <   - 2*sig(I) ...         27 Check
```

---

0 **ALERT level A** = Most likely a serious problem - resolve or explain
0 **ALERT level B** = A potentially serious problem, consider carefully
6 **ALERT level C** = Check. Ensure it is not caused by an omission or oversight
20 **ALERT level G** = General information/check it is not something unexpected

4 ALERT type 1 CIF construction/syntax error, inconsistent or missing data
7 ALERT type 2 Indicator that the structure model may be wrong or deficient
7 ALERT type 3 Indicator that the structure quality may be low
6 ALERT type 4 Improvement, methodology, query or suggestion
2 ALERT type 5 Informative message, check

It is advisable to attempt to resolve as many as possible of the alerts in all categories. Often the minor alerts point to easily fixed oversights, errors and omissions in your CIF or refinement strategy, so attention to these fine details can be worthwhile. In order to resolve some of the more serious problems it may be necessary to carry out additional measurements or structure refinements. However, the purpose of your study may justify the reported deviations and the more serious of these should normally be commented upon in the discussion or experimental section of a paper or in the "special_details" fields of the CIF. checkCIF was carefully designed to identify outliers and unusual parameters, but every test has its limitations and alerts that are not important in a particular case may appear. Conversely, the absence of alerts does not guarantee there are no aspects of the results needing attention. It is up to the individual to critically assess their own results and, if necessary, seek expert advice.

Publication of your CIF in IUCr journals

A basic structural check has been run on your CIF. These basic checks will be run on all CIFs submitted for publication in IUCr journals (*Acta Crystallographica*, *Journal of Applied Crystallography*, *Journal of Synchrotron Radiation*); however, if you intend to submit to *Acta Crystallographica Section C* or *E* or *IUCrData*, you should make sure that full publication checks are run on the final version of your CIF prior to submission.

Publication of your CIF in other journals

Please refer to the *Notes for Authors* of the relevant journal for any special instructions relating to CIF submission.

PLATON version of 28/11/2022; check.def file version of 28/11/2022

Datablock I - ellipsoid plot

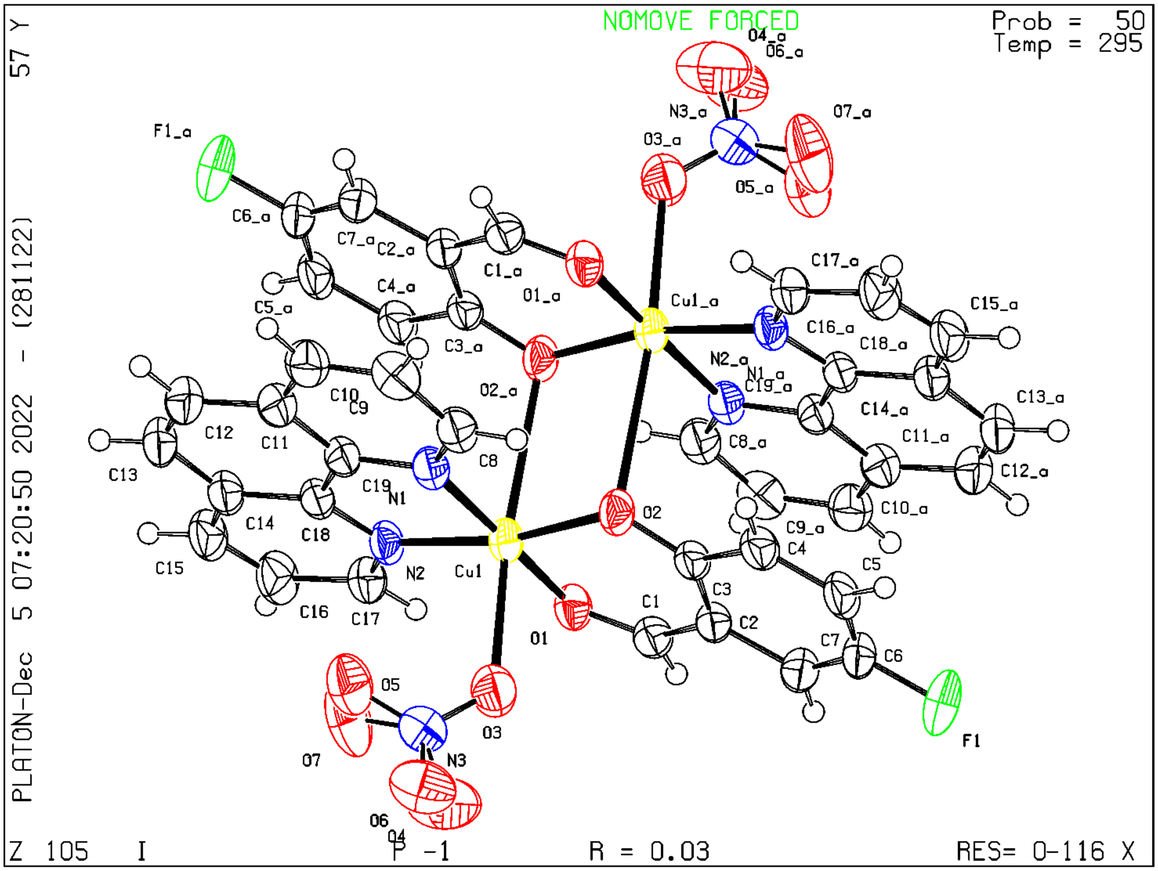

Supplement: Supplementary file 1 [file molecules-27-08929-s001.zip › checkcif of 4.pdf]
