# Supplementary material for: Copper(II) Complexes of 5–Fluoro–Salicylaldehyde: Synthesis, Characterization, Antioxidant Properties, Interaction with DNA and Serum Albumins
Source: Molecules. 2022 Dec 15;27(24):8929. doi: 10.3390/molecules27248929 (PMC9782626; doi:10.3390/molecules27248929)
Supplement: Supplementary file 1 [file molecules-27-08929-s001.zip › molecules-2062742, ESI revised.pdf]

Copper(II) complexes of 5–fluoro–salicylaldehyde: Synthesis, characterization, antioxidant properties, interaction with DNA and serum albumins

Zisis Papadopoulos,[1,a] Efstratia Doulopoulou,[1,a] Ariadni Zianna,[1] Antonios G. Hatzidimitriou,[1] George Psomas,[1,*]

[1] *Laboratory of Inorganic Chemistry, Department of Chemistry, Aristotle University of Thessaloniki, Thessaloniki GR–54124, GREECE.;*

[a] These authors contributed equally.

* Correspondence: gepsomas@chem.auth.gr

Supplementary material

CONTENT	Page
**S1** Antioxidant activity assay	3
**S1.1** Determination of the reducing activity of the radical DPPH	3
**S1.2** Assay of radical cation ABTS-scavenging activity	3
**S1.3** Reduction of hydrogen peroxide	3
**S2** Binding studies with CT DNA	4
**S2.1** Binding study with CT DNA by UV-vis spectroscopy	4
**S2.2** CT DNA-binding studies by viscosity measurements	4
**S2.3** EB-displacement studies	4
**S3** Albumin-binding studies	5
**S4** References	6
**Figure S1.** IR spectra of the complexes	7
**Figure S2.** UV-vis spectra of the complexes	9

S1 Antioxidant activity assay

The antioxidant activity of the compounds was evaluated *via* their ability to scavenge *in vitro* free radicals such as DPPH and ABTS and to reduce H_2O_2. All the experiments were carried out at least in triplicate and the standard deviation of absorbance was less than 10% of the mean.

S1.1 Determination of the reducing activity of the radical DPPH

To an ethanolic solution of DPPH (0.1 mM) an equal volume solution of the compounds (0.1 mM) in ethanol was added. Absolute ethanol was also used as control solution. The absorbance at 517 nm was recorded at room temperature after 30 and 60 min, in order to examine the possible existence of a potential time-dependence of the DPPH radical scavenging activity [S1]. The DPPH-scavenging activity of the compounds was expressed as the percentage reduction of the absorbance values of the initial DPPH solution (DPPH%). NDGA and BHT were used as reference compounds.

S1.2 Assay of radical cation ABTS-scavenging activity

The ABTS assay was performed to determine the activity of the compounds to scavenge the radical cation ABTS. Initially, a water solution of ABTS was prepared (2 mM). ABTS radical cation ($ABTS^{+\cdot}$) was produced by the reaction of ABTS stock solution with potassium persulfate (0.17 mM) and the mixture was stored in the dark at room temperature for 12-16 h before its use. The ABTS was oxidized incompletely because the stoichiometric reaction ratio of ABTS and potassium persulfate is 1:0.5. The absorbance became maximal and stable only after more than 6 h of reaction although the oxidation of the ABTS started immediately. The radical was stable in this form for more than 2 days when allowed to stand in the dark at room temperature. Afterwards, the $ABTS^{+\cdot}$ solution was diluted in ethanol to an absorbance of 0.70 at 734 nm and 10 μL of diluted compounds or standards (0.1 mM) in DMSO were added. The absorbance was recorded out exactly 1 min after initial mixing [S1]. The ABTS radical scavenging activity was expressed as the percentage inhibition of the absorbance of the initial ABTS solution (ABTS%). Trolox was used as an appropriate standard.

S1.3 Reduction of hydrogen peroxide

The ability of the compounds to reduce hydrogen peroxide (H_2O_2) was estimated according to the method described in the literature [S2]. The reaction mixture contained 20 μL of each of the tested compounds (0.1 mM) and 5 μL H_2O_2 solution (40 mM) in phosphate buffer (50 mM, pH = 7.4). The absorbance was measured at 230 nm after 10 min. The antioxidant activity (reduction of hydrogen peroxide) of the compounds was

expressed as the percentage decrease of the initial H_2O_2 solution (H_2O_2%). L-ascorbic acid (or vitamin C) was used as a standard.

S2 Binding studies with CT DNA

The interaction of the compounds with CT DNA was studied by UV-vis spectroscopy, viscosity measurements and *via* competitive studies with EB by fluorescence emission spectroscopy.

S2.1 Binding study with CT DNA by UV-vis spectroscopy

The interaction of the compounds with CT DNA has been studied by UV-vis spectroscopy in order to investigate the possible binding modes to CT DNA and to calculate the DNA-binding constants (K_b). The K_b constants (in M^{-1}) were determined by the Wolfe-Shimer equation (eq. S1) [S3] and the plots [DNA]/(ε_A-ε_f) *versus* [DNA] using the UV-vis spectra of the compounds (20-100 μM) recorded for a constant concentration with increasing concentrations of CT DNA for diverse [complex]/[DNA] mixing ratios (= *r*). Control experiments with DMSO were performed and no changes in the spectra of CT DNA were observed. According to the Wolfe-Shimer equation (eq. S1):

$$\frac{[\mathrm{DNA}]}{(\varepsilon_A - \varepsilon_f)} = \frac{[\mathrm{DNA}]}{(\varepsilon_b - \varepsilon_f)} + \frac{1}{K_b(\varepsilon_b - \varepsilon_f)} \qquad \text{(eq. S1)}$$

where [DNA] is the concentration of DNA in base pairs, $\varepsilon_A = A_{obsd}$/[compound], ε_f = the extinction coefficient for the free compound and ε_b = the extinction coefficient for the compound in the fully bound form. K_b is given by the ratio of slope to the y intercept in plots [DNA]/(ε_A-ε_f) *versus* [DNA].

S2.2 CT DNA-binding studies by viscosity measurements

The interaction of compounds with DNA has been evaluated *via* the study of the CT DNA viscosity ([DNA] = 0.1 mM) in a buffer solution (150 mM NaCl and 15 mM trisodium citrate at pH 7.0) in the presence of increasing amount of complexes (up to the value of *r* = 0.36). The obtained data are presented as $(\eta/\eta_0)^{1/3}$ *versus r*, where η is the viscosity of DNA in the presence of the compound, and η_0 is the viscosity of DNA alone in buffer solution.

S2.3 EB-displacement studies

The competition of the complexes with EB was investigated by fluorescence emission spectroscopy in order to examine whether the compounds can displace EB from its DNA-EB conjugate. The CT DNA-EB complex was formed by pre-treating 20 μM EB and 26 μM CT DNA in buffer (150 mM NaCl and 15 mM trisodium citrate at pH = 7.0). The possible displacement of EB by the compounds and subsequently their intercalating effect was studied by the stepwise addition of a certain amount of the solution of each

compound into the solution of the CT DNA-EB adduct. The solutions were excited at 540 nm and the emission was monitored from 550-700 nm with λ_{max}= 592-595 nm and the effect of the addition of each compound to the CT-DNA EB solution was recorded. The compounds do not display any fluorescence emission bands at room temperature in solution or in the presence of CT DNA or EB under the same experimental conditions (λ_{ex} = 540 nm); therefore, the observed quenching of the EB-DNA solution may be attributed to the displacement of EB from its EB-DNA adduct.

The Stern-Volmer constants (K_{SV}, in M^{-1}) were calculated according by the linear Stern-Volmer equation (eq. S2) [S4] and the respective plots Io/I *versus* [compound].

$$\frac{Io}{I} = 1 + k_q\tau_0[Q] = 1 + K_{SV}[Q] \qquad \text{(eq. S2)}$$

where Io and I are the emission intensities of the EB-DNA solution in the absence and the presence of the compounds, respectively, τ_o= the average lifetime of the emitting system without the quencher and kq = the quenching constant. Taking τ_o = 23 ns as the fluorescence lifetime of the EB-DNA adduct [S5], the quenching constants (k_q, in $M^{-1}s^{-1}$) of the compounds were calculated according to eq. S3 [S4]:

$$K_{SV} = k_q\tau_o \qquad \text{(eq. S3)}$$

S3 Albumin-binding studies

In order to investigate if the compounds can bind to carrier proteins like serum albumins, we carried out albumin binding study by tryptophan fluorescence quenching experiments using bovine serum albumin (BSA, 3 μM) in buffer (containing 15 mM trisodium citrate and 150 mM NaCl at pH 7.0). The quenching of the emission intensity of tryptophan residues of BSA at 343 nm was monitored using the compounds as quenchers with increasing concentration [S4]. The fluorescence emission spectra of the compounds were also recorded with λ_{ex} = 295 nm. No fluorescence emission band was recorded and thus it was not related to the compounds. The influence of the inner-filter effect [S6] on the measurements was evaluated by equation S4.

$$I_{corr} = I_{meas} \times 10^{\frac{\varepsilon(\lambda_{exc})cd}{2}} \times 10^{\frac{\varepsilon(\lambda_{em})cd}{2}} \qquad \text{(eq. S4)}$$

Where I_{corr} = corrected intensity, I_{meas} = the measured intensity, c = the concentration of the quencher, d = the cuvette (1 cm), $\varepsilon_{(\lambda exc)}$ and $\varepsilon_{(\lambda em)}$ = the ε of the quencher at the excitation and the emission wavelength, respectively, as calculated from the UV-vis spectra of the compound [S6].

The Stern-Volmer and Scatchard graphs are used to study the interaction of the compounds with serum albumins. According to Stern-Volmer quenching equation (eq. S2), where Io = the initial tryptophan fluorescence intensity of SA, I = the tryptophan fluorescence intensity of SA after the addition of the quencher, k_q = the quenching

constant, K_{SV} = the Stern-Volmer constant, τ_o = the average lifetime of SA without the quencher, and, taking as fluorescence lifetime (τ_o) of tryptophan in SA at around 10^{-8} s [S4], K_{SV} (in M^{-1}) can be obtained by the slope of the diagram Io/I *versus* [compound] (Stern-Volmer plots), and subsequently the quenching (k_q, in $M^{-1}s^{-1}$) may be calculated from eq. S3.

From the Scatchard equation (eq. S5):

$$\frac{\Delta I/Io}{[Q]} = nK - K\frac{\Delta I}{Io} \qquad \text{(eq. S5)}$$

where n is the number of binding sites per albumin and K is the SA-binding constant (K, in M^{-1}) is calculated from the slope in plots (ΔI/Io)/[complex] *versus* ΔI/Io and n is given by the ratio of y intercept to the slope [S7].

S4 References

S1 Kontogiorgis, C.; Hadjipavlou–Litina, D. Biological Evaluation of Several Coumarin Derivatives Designed as Possible Anti–inflammatory/Antioxidant Agents. *J. Enz. Inhib. Med. Chem.*, **2003**, 180, 63–69.

S2 Ruch, R.J.; Cheng, C.; Klaunig, J.E. Prevention of cytotoxicity and inhibition of intercellular communication by antioxidant catechins isolated from Chinese green tea. *Carcinogenesis*, **1989**, 10, 1003–1008.

S3 Wolfe, A.; Shimer, G.; Meehan, T. Polycyclic Aromatic Hydrocarbons Physically Intercalate into Duplex Regions of Denatured DNA. *Biochemistry* **1987**, *26*, 6392–6396.

S4 Lakowicz, J.R. *Principles of Fluorescence Spectroscopy*, 3rd ed.; Plenum Press: New York, NY, USA, **2006.**

S5 Heller, D.P.; Greenstock, C.L. Fluorescence lifetime analysis of DNA intercalated ethidium bromide and quenching by free dye. *Biophys. Chem.* **1994**, *50*, 305–312.

S6 Stella, L.; Capodilupo, A.L.; Bietti, M. A reassessment of the association between azulene and [60]fullerene. Possible pitfalls in the determination of binding constants through fluorescence spectroscopy. *Chem. Commun.* **2008**, 39, 4744–4746.

S7 Wang, Y.; Zhang, H.; Zhang, G.; Tao, W.; Tang, S. Interaction of the flavonoid hesperidin with bovine serum albumin: A fluorescence quenching study. *J. Luminescence*, **2007**, 126, 211–218.

(A)	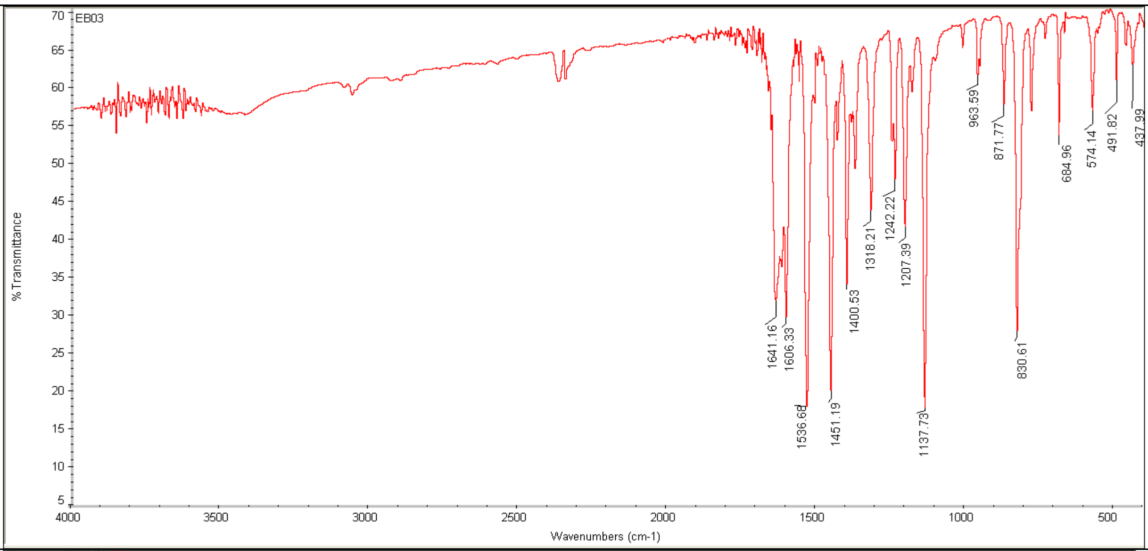
(B)	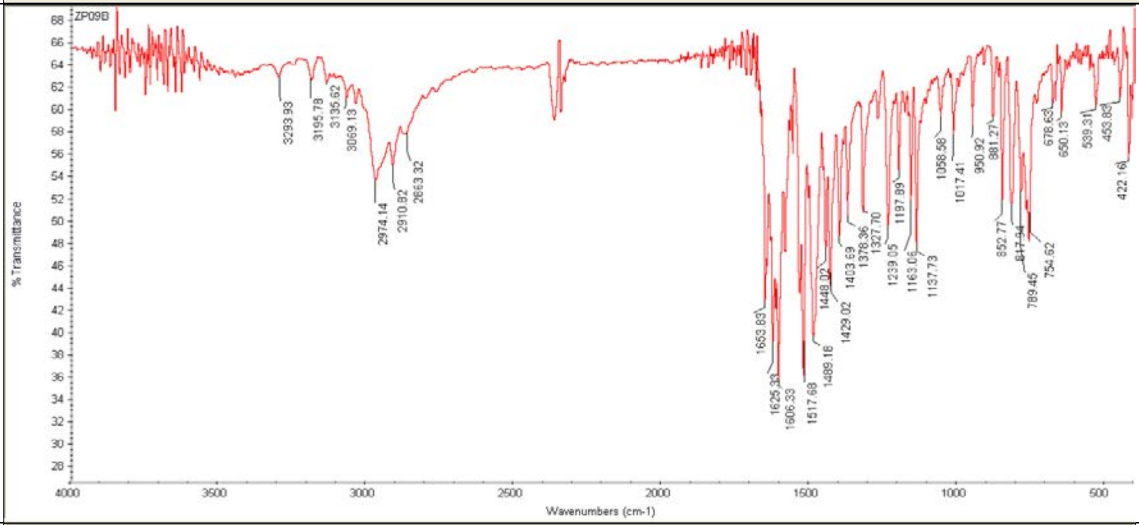
(C)	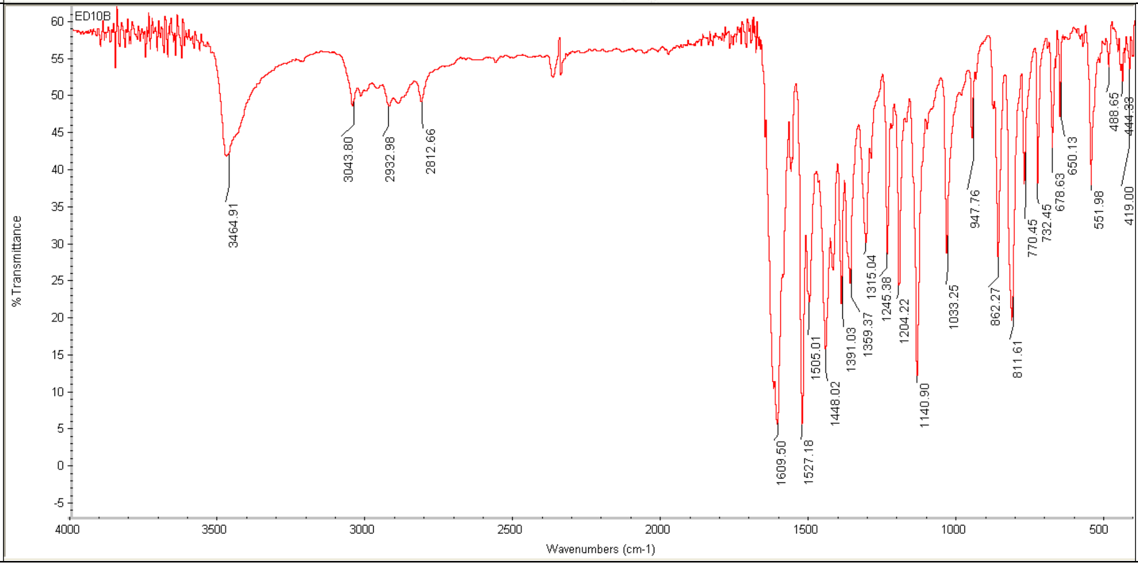

EB03
% Transmittance
Wavenumbers (cm-1)
1641.16
1606.33
1536.68
1451.19
1400.53
1318.21
1242.22
1207.39
1137.73
963.59
871.77
830.61
684.96
574.14
491.82
437.99
ZP09B
3293.93
3195.78
3135.62
3069.13
2974.14
2910.82
2863.32
1653.83
1625.33
1606.33
1517.68
1489.18
1448.02
1429.02
1403.69
1378.36
1327.70
1239.05
1197.89
1163.06
1137.73
1058.58
1017.41
950.92
881.27
852.77
817.94
789.45
754.62
678.63
650.13
539.31
453.83
422.16
ED10B
3464.91
3043.80
2932.98
2812.66
1609.50
1527.18
1505.01
1448.02
1391.03
1359.37
1315.04
1245.38
1204.22
1140.90
1033.25
947.76
862.27
811.61
770.45
732.45
678.63
650.13
551.98
488.65
444.33
419.00

(D)	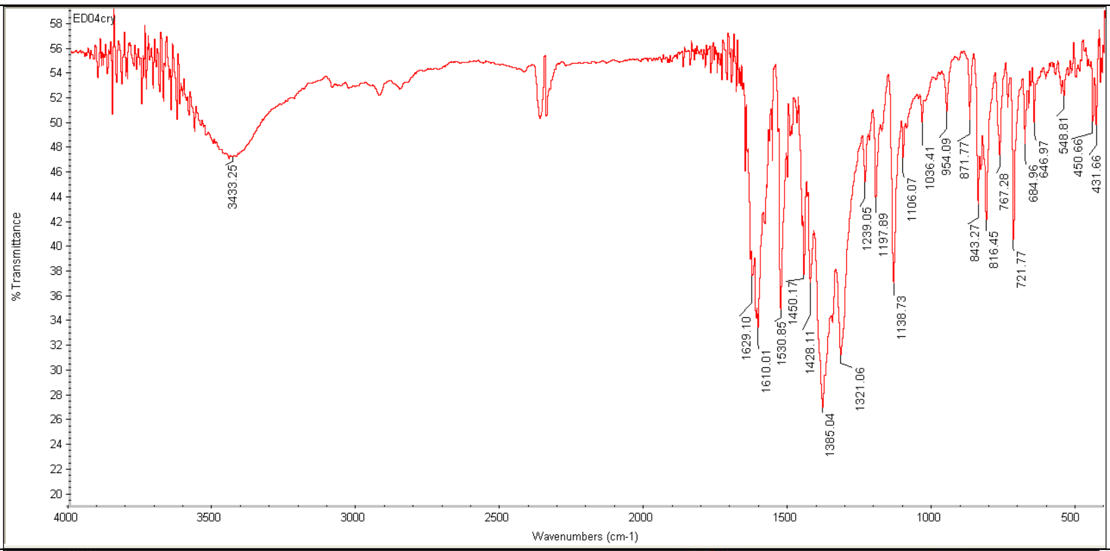
(E)	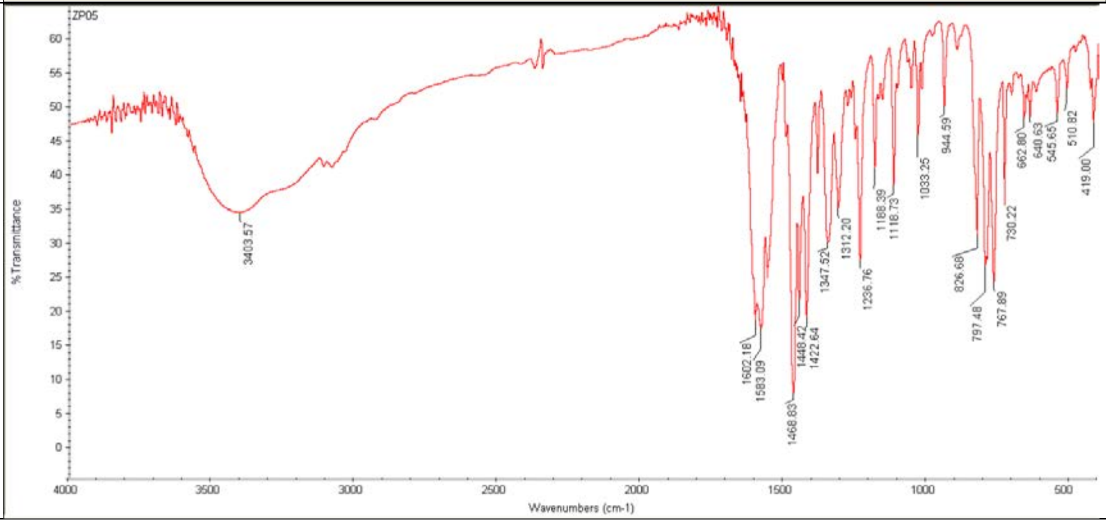

Figure S1. IR spectrum of (A) complex [Cu(5–F–salo)$_2$], **1,** (B) complex [Cu(5–F–salo)(bipyam)Cl]**, 2, (C)** complex [Cu(5–F–salo)(neoc)Cl]·CH_3OH **(3),** (D) complex [Cu(5–F–salo)(phen)(NO_3)], **4** and **(E)** complex [Cu(5–F–salo)(bipy)(NO_3)], **5.**

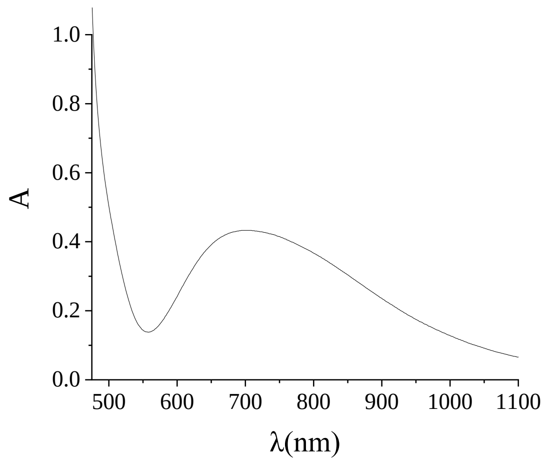

(A)

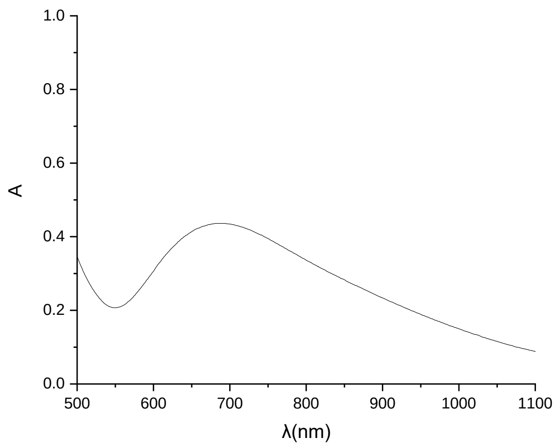

(B)

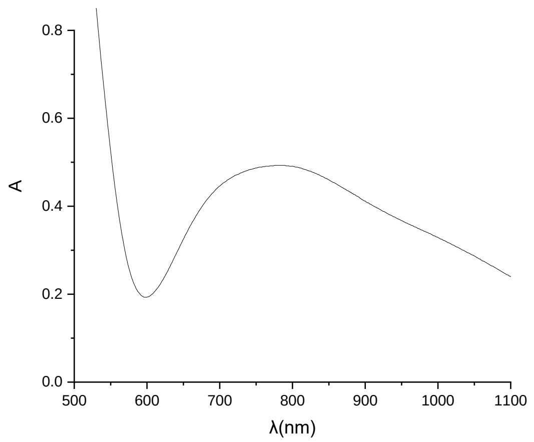

(C)

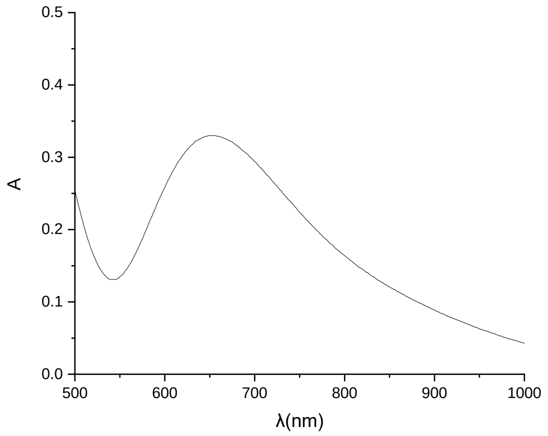

(D)

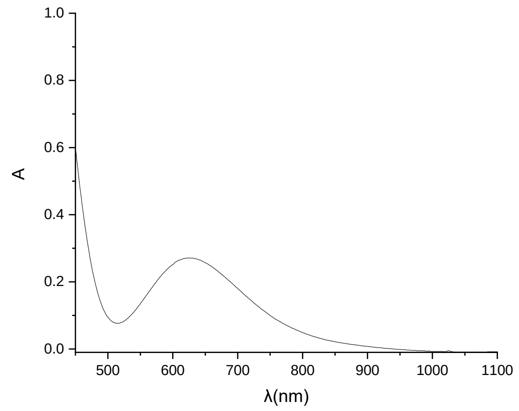

(E)

Figure S2. UV-vis spectra in DMSO (5 mM) of (A) complex [Cu(5–F–salo)$_2$], **1,** (B) complex [Cu(5–F–salo)(bipyam)Cl]**, 2, (C)** complex [Cu(5–F–salo)(neoc)Cl]·CH_3OH **(3),** (D) complex [Cu(5–F–salo)(phen)(NO_3)], **4** and **(E)** complex [Cu(5–F–salo)(bipy)(NO_3)], **5.**
